# Differential expression analysis identifies a prognostically significant extracellular matrix–enriched gene signature in hyaluronan-positive clear cell renal cell carcinoma

**DOI:** 10.1038/s41598-024-61426-3

**Published:** 2024-05-09

**Authors:** Otto Jokelainen, Teemu J. Rintala, Vittorio Fortino, Sanna Pasonen-Seppänen, Reijo Sironen, Timo K. Nykopp

**Affiliations:** 1 Institute of Clinical Medicine, Pathology and Forensic Medicine, University of Eastern Finland, Kuopio Campus, P.O. Box 1627, 70211 Kuopio, Finland; 2 Department of Clinical Pathology, Kuopio University Hospital, Kuopio, Finland; 3 Institute of Biomedicine, University of Eastern Finland, Kuopio, Finland; 4 Department of Surgery, Kuopio University Hospital, Kuopio, Finland; 5 Institute of Clinical Medicine, Surgery, University of Eastern Finland, Kuopio, Finland

**Keywords:** Medical research, Urology, Renal cancer, Prognostic markers

## Abstract

Hyaluronan (HA) accumulation in clear cell renal cell carcinoma (ccRCC) is associated with poor prognosis; however, its biology and role in tumorigenesis are unknown. RNA sequencing of 48 HA-positive and 48 HA-negative formalin-fixed paraffin-embedded (FFPE) samples was performed to identify differentially expressed genes (DEG). The DEGs were subjected to pathway and gene enrichment analyses. The Cancer Genome Atlas Kidney Renal Clear Cell Carcinoma (TCGA-KIRC) data and DEGs were used for the cluster analysis. In total, 129 DEGs were identified. HA-positive tumors exhibited enhanced expression of genes related to extracellular matrix (ECM) organization and ECM receptor interaction pathways. Gene set enrichment analysis showed that epithelial–mesenchymal transition-associated genes were highly enriched in the HA-positive phenotype. A protein–protein interaction network was constructed, and 17 hub genes were discovered. Heatmap analysis of TCGA-KIRC data identified two prognostic clusters corresponding to HA-positive and HA-negative phenotypes. These clusters were used to verify the expression levels and conduct survival analysis of the hub genes, 11 of which were linked to poor prognosis. These findings enhance our understanding of hyaluronan in ccRCC.

## Introduction

Global Cancer Statistics showed that approximately 430,000 new cases of kidney cancer (KC) were diagnosed in 2020, accounting for 2.2% of all human malignancies. It contributed to 180,000 deaths worldwide, making it the most lethal urological malignancy worldwide^1^, and the incidence of KC is increasing^2^. Renal cell carcinoma (RCC) is the most common type of kidney cancer, accounting for > 90% of primary kidney tumors^3^. The three most common histological subtypes of RCC are clear cell RCC (ccRCC), papillary RCC, and chromophobe RCC. Recently, more entities with molecularly defined pathogenesis have been identified^4^. RCC is often diagnosed incidentally, and one-third of the patients present with metastatic disease. Twenty percent of patients who undergo surgery for a primary tumor later develop metastases. Despite recent advances in systemic therapies, the prognosis of metastatic disease remains dismal^5,6^. Therefore, it is imperative to identify new biomarkers for disease detection, prognostication, and treatment.

Hyaluronan (HA) is a ubiquitous large glycosaminoglycan (GAG) found in the extracellular matrix (ECM), where it forms a pericellular coat surrounding cells and functions as a cushion^7^. It is composed of a variable number of repeating disaccharide units of N-acetyl-glucosamine (GlcNAc) and glucuronic acid (GlcUA), with an average molecular mass ranging from 1000 to 8000 kD^8^. The turnover of hyaluronan is rapid, and one-third of the hyaluronan mass undergoes turnover daily^9^. Hyaluronan is synthesized by the hyaluronan synthase enzymes HAS1, HAS2, and HAS3^10^. Degradation is mediated mainly by the family of hyaluronidases (HYAL 1-4, PH20, CEMIP, and TMEM2)^11–13^. In addition to RCC, increased HA content has been associated with worse outcomes and more aggressive tumor growth in several human malignancies, including breast cancer, colon carcinoma, gastric carcinoma, thyroid cancer, pancreatic cancer, lung adenocarcinoma, lymphoma, hepatocellular carcinomas, and gliomas^14–23^. It is postulated that HA acts as a barrier that shields tumor cells from insults, such as therapeutic agents and the immune system, and could serve as a potential target for anticancer drugs^24^. The use of PEGylated human hyaluronidase (PEGPH20) has shown promising efficacy in sensitizing pancreatic cancer cells to radiotherapy and in improving the efficacy of anti-PD-1 therapy^25,26^.

In normal human kidneys, most HA is produced in the renal medulla, while the renal cortex, from which renal cell carcinomas usually arise, is hyaluronan poor^27^. Increased cortical HA content is associated with various non-neoplastic kidney diseases/conditions such as acute kidney injury, chronic kidney disease, allograft, and diabetic nephropathy^28–31^. To date, reports on HA in RCCs are limited. In our previous study, we showed that increased cellular hyaluronan conveys poor prognosis in patients with ccRCC^14^. In addition, a higher hyaluronan content was associated with a higher tumor grade. The individual molecules associated with HA have been studied on gene expression levels. Chi et al.^32^ showed that expression of HAS1 was increased in RCC tissue compared with adjacent normal tissue while HYAL1 expression was lower in ccRCC than in normal renal tissue^32^. Cai et al. found that HAS1-3 mRNA expression was higher in human ccRCC tissues than in adjacent normal tissues^33^. However, only HAS3 protein expression was higher. In conclusion, the results of expression studies are inconsistent, and the expression levels of different HA family proteins might not necessarily reflect overall HA levels. Therefore, to better understand the biological background of hyaluronan accumulation in ccRCC, we performed RNA sequencing of previously found hyaluronan-positive and-negative tumor cohorts. The aim of this study was to investigate the differences in RNA expression profiles and to find new potential hyaluronan-associated molecules.

## Materials and methods

### Patients and sample selection

**Figure 1:**
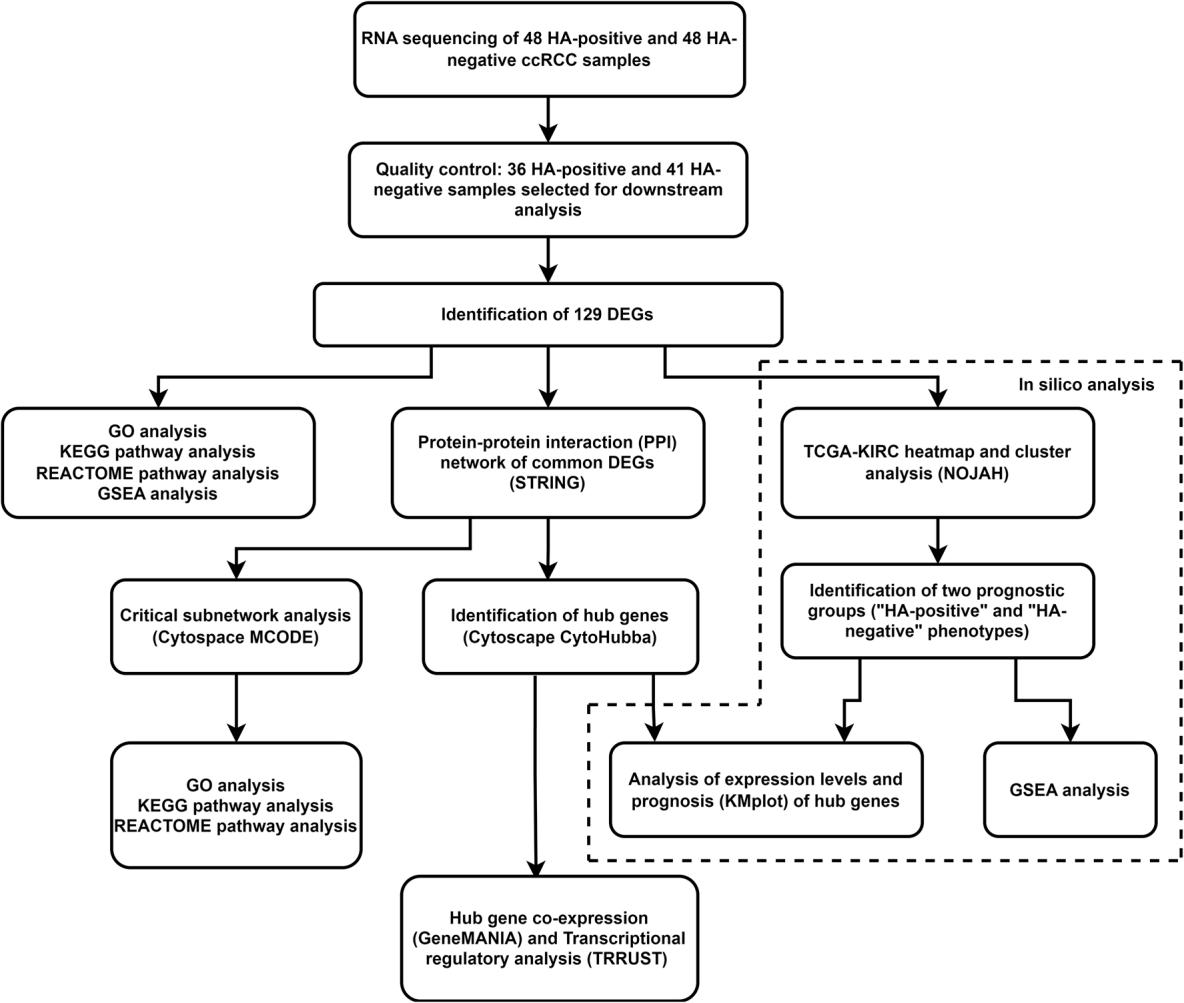
Research flowchart. The dashed line represents in silico analysis using the Cancer Genome Atlas Kidney Renal Clear Cell Carcinoma (TCGA-KIRC) data. *HA* Hyaluronan, *ccRCC* clear cell renal cell carcinoma, *DEG* differentially expressed gene, *GO* Gene Ontology, *KEGG* Kyoto Encyclopedia of Genes and Genomes, *GSEA* Gene Set Enrichment Analysis, *STRING* Search Tool for the Retrieval of Interacting Genes/Proteins, *TRRUST* Transcriptional Regulatory Relationships Unraveled by Sentence-based Text mining, *NOJAH* NOt Just Another Heatmap.

A research flowchart of this study is shown in Fig. 1. Formalin-fixed and paraffin-embedded (FFPE) tissue samples from patients who underwent surgery for ccRCC in the period 2000–2013 at Kuopio University Hospital were collected from the Biobank of Eastern Finland. The study (Hyaluronan in Renal Cell Carcinoma, HARCC) was approved by the Ethics Committee of the Northern Savo Hospital District (379/2016, November 1st, 2016). The diagnostic samples were processed and diagnosed according to the routine protocol in the Department of Clinical Pathology. Hyaluronan staining and evaluation were performed as described by Jokelainen et al.^14^. We selected 48 hyaluronan-positive and 48 hyaluronan-negative tumor samples for RNA sequencing on the basis of tumor grade, sarcomatoid change, sex, survival, and metastasis status (Table 1). Three 1-mm-wide tissue cores were punched from each representative tumor block.

**Table 1:** Characteristics of 96 renal cell carcinoma tissue samples.

	Hyaluronan-positive N (%)		Hyaluronan-negative N (%)	
Samples	48		48	
Sex				
Male	31	(64.6)	24	(50)
Female	17	(35.4)	24	(50)
Age (mean, range)	61.8	(41–82)	67.0	(36–86)
WHO/ISUP grade				
1	2	(4.2)	8	(16.7)
2	22	(45.8)	22	(45.8)
3	10	(20.8)	9	(18.8)
4	14	(29.2)	9	(18.8)
Sarcomatoid change				
No	41	(85.4)	43	(89.6)
Yes	7	(14.6)	5	(10.4)
Disease-related death				
No	28	(58.6)	35	(72.9)
Yes	20	(41.2)	13	(27.1)
Clinical stage				
I	16	(33.3)	24	(50.0)
II	9	(18.8)	8	(16.7)
III	12	(25.0)	7	(14.6)
IV	11	(22.9)	9	(18.8)
Metastasis at diagnosis				
M0	37	(77.1)	39	(81.3)
M1	11	(22.9)	9	(18.7)

### Next-generation sequencing

RNA was isolated by use of an RNeasy FFPE Kit (Qiagen), and deparaffinization was performed using 640–750 µL deparaffinization solution from the kit with 60–90 min incubation at 56 °C. RNA was eluted with 2′ 14 µL of RNase-free water. rRNA was removed by use of a QIASeq FastSelect (rRNA HMR, Qiagen), using 1 µg RNA or as much as could be handled by the kit (max 10 µL). RNA-sequencing libraries were constructed with a TruSeq Stranded mRNA Library Prep kit (Illumina) and 0.3 µL adapters with 30 PCR cycles. Barcodes were optimized by use of BARCOSEL software (http://ekhidna2.biocenter.helsinki.fi/barcosel/)^34^. Sequencing was performed using an Illumina Novaseq 6000 instrument. Adapter sequences, low-quality bases (q = 25), and short sequences (m = 30) were first trimmed using cutadapt (v.4.1)^35^ (https://cutadapt.readthedocs.io/en/stable/). The fourteenth (p14) patch release for the GRCh38 reference assembly and annotation was downloaded from https://ftp.ncbi.nlm.nih.gov/genomes/all/GCF/000/001/405/GCF_000001405.40_GRCh38.p14/. The STAR aligner (v2.7.9a_2021-06-25) (https://code.google.com/archive/p/rna-star/) with default parameters was used to map reads against the reference sequence^36^. Sorting and indexing of the alignment files were performed using Samtools (v.1.10) (https://www.htslib.org/)^37^.

### Quality control and differentially expressed gene analysis

A significant portion of the mapped reads was concentrated on only a few genes in each sample, which necessitated an additional filtering step using Samtools (v. 1.16.1) to mark duplicated reads for removal using the no-multi-dup option. Gene counts were computed by use of R (v.4.1.1) (https://www.r-project.org/) and Rsubread (v.2.6.4) (https://bioconductor.org/packages/release/bioc/html/Rsubread.html) with multi-mapping and multi-overlapping reads removed^38,39^. In addition to the read-level quality control (QC) detailed in section "next-generation sequencing", further QC steps were performed according to the recommendations specified by Liu et al.^40^ for FFPE RNA-seq count data^40^. As FFPE samples were used, the total number of mapped reads in all samples was generally low, as expected. Therefore, the quality metric used for filtering the samples was the median sample-sample Spearman correlation after count normalization. Samples with a median correlation below 0.75 were removed, leaving 36 hyaluronan-positive and 41 hyaluronan-negative samples. Differential expression analysis was performed using edgeR (v.3.34.1) (https://bioconductor.org/packages/release/bioc/html/edgeR.html)^41^. Only protein-coding genes and genes with mean counts above 1 across all samples were included, leaving 10,633 genes for consideration. Count normalization was performed using the trimmed mean M-value method in edgeR. The results we plotted using the ggplot2 package (v.3.4.2) (https://ggplot2.tidyverse.org)^42^.

### Gene Ontology and pathway enrichment analysis

Gene Ontology (GO) is a bioinformatics database established to provide simple annotation of gene products^43,44^. GO terms include biological processes (BP), cellular components (CC), and molecular functions (MF) of gene products. The Kyoto Encyclopedia of Genes and Genomes (KEGG) and REACTOME are free online databases containing information on biological pathways, molecular interactions, and reactions^45–48^. Database analysis was performed to investigate the molecular function of the identified DEGs using ToppGene (https://toppgene.cchmc.org/enrichment.jsp)^49^. ToppGene is a bioinformatics portal for gene-list enrichment analysis. The cutoff value for the false discovery rate (FDR) was set at p < 0.05. The Benjamini–Hochberg procedure was used to account for multiple testing. Results were plotted by SRplot (https://www.bioinformatics.com.cn/srplot), an online platform for data analysis and visualization^50^.

### Gene set enrichment analysis

Gene Set Enrichment Analysis (GSEA) was performed to examine the gene expression profiles of hyaluronan-positive and hyaluronan-negative samples which had passed quality control steps^51,52^. The hyaluronan-positive phenotype was compared with the hyaluronan-negative phenotype. The trimmed mean of M values (TMM)-normalized count data and phenotype data were uploaded to GSEA software (build v.4.3.2.) (https://www.gsea-msigdb.org/gsea/index.jsp) and Human MSigDB h.all.v2023.1.Hs.symbols hallmark gene set was chosen. The number of permutations was set to 1000. All other run parameters were maintained at their default values.

### Protein-to-protein network construction and subnetworks

The Search Tool for the Retrieval of Interacting Genes/Proteins (STRING) is a biological database of known and predicted protein–protein interactions (PPI) (https://string-db.org/)^53^. This includes the interactions derived from experiments and computationally predicted interactions. STRING (v.11.5) was used to predict interactions between the DEGs. Interactions with a combined score > 0.4 were considered statistically significant. The PPI network provided by STRING was imported into and visualized in Cytoscape (v.3.9.1.) (http://www.cytoscape.org)^54^. The Cytoscape plug-in MCODE (v.2.0.2) was used to identify highly interconnected regions in the network^55^. Settings used were as follows: Cluster Finding Haircut, Node Score Cutoff = 0.2, K-Core = 2, Max. Depth = 100.

### Hub genes discovery and analysis

Hubgene analysis was performed using the CytoHubba (v.0.1) plug-in in Cytoscape^56^. Seven common algorithms (MCC, MNC, Degree, Closeness, Radiality, Stress, and EPC) were used to identify hub genes, and the UpSet intersection plot (by SRPlot) was used to identify common genes. The hub genes were then exported to GeneMANIA (http://www.genemania.org/), a predictor software used to identify other genes related to a set of input genes and internal associations in the gene sets^57^. Default methods were used to calculate connection weights. Lastly, we used Transcriptional Regulatory Relationships Unraveled by Sentence-based Text Mining (TRRUST) (v.2) to predict transcription factors (TFs) of hub genes (https://www.grnpedia.org/trrust/)^58^. TFs with adjusted P-value < 0.05 were considered significant. Subsequently, we used the TCGA-KIRC dataset to examine the expression of these TFs^59^.

### In-silico TCGA heatmap and cluster analysis

To test the gene signature identified by DEG analysis with another dataset, the TCGA-KIRC RNA expression dataset was downloaded via the Bioconductor package TCGABiolinks (v.2.26.0) (https://bioconductor.org/packages/release/bioc/html/TCGAbiolinks.html) using R (v.4.2.2) and RStudio (2022.12.0 + 353)^38,60,61^. Phenotype data concerning tumor grade and patient survival were downloaded from the same source. Data from 537 ccRCC samples were collected after removing duplicates. The count values were TMM-normalized and log2(count + 1)-transformed. Genome-Wide Heatmap (GWH) analysis was performed using the NOJAH (NOt Just Another Heatmap) (v.1) interactive tool (https://github.com/bbisr-shinyapps/NOJAH/)^62^. A heatmap was plotted, using the set of 129 DEGs. The values selected for hierarchical clustering analysis parameters were the Z-score method for data normalization type, Euclidean for the distance method, and ward.D2 for the clustering method. Tumor phenotype data were combined into a heatmap, using NOJAH. Furthermore, the methylation status of TCGA samples, as described by Ricketts et al.^63^, was combined with the heatmap^63^. The observed distribution of tumor grade, patient survival, and methylation cluster within each heatmap cluster was tested using the chi-square test.

### TCGA GSEA analysis

GSEA analysis was performed on TCGA-KIRC data using the newly discovered HA-positive and HA-negative phenotypes. TMM-normalized expression and phenotype data were uploaded to GSEA software (build v.4.3.2.) and Human MSigDB h.all.v2023.1.Hs.symbols hallmark gene set was chosen. The number of permutations was set to 1000. All other run parameters were maintained at their default values.

### Hub gene expression analysis in silico

The mRNA expression levels of the identified hub genes were investigated, using the TCGA-KIRC dataset. RNA-seq data from 72 healthy renal tissues were downloaded using the TCGAbiolinks package (v.2.26.0) in R (v.4.2.2). TCGA samples identified belonging to “HA-negative” and “HA-positive” gene expression clusters were compared against each other and to normal renal tissue. The expression levels of the hub genes were TPM-normalized and log2-transformed for each cohort. The Mann–Whitney U-test was used to compare each group with the other two groups, and the expression levels were box-plotted using R package ggpubr (v.0.6.0) (https://rpkgs.datanovia.com/ggpubr/)^64^.

### Prognostic value of hub genes

We used the Kaplan–Meier Plotter online database (http://kmplot.com/analysis/) containing TCGA-KIRC data from 530 patients to analyze the prognosis of hub genes^65^. Kaplan–Meier estimators were plotted, and hazard ratios were calculated for overall survival (OS) and disease-free survival (DFS). The samples were stratified into low- and high-expression groups based on the median cut-off (50%). Statistical significance was set at p < 0.05 and HR > 1.0 were considered significant.

### Ethical considerations

The study was conducted in accordance with the Declaration of Helsinki and approved by the Ethics Committee of the Northern Savo Hospital District (379/2016, November 1st, 2016).

## Results

### DEG identification

**Figure 2:**
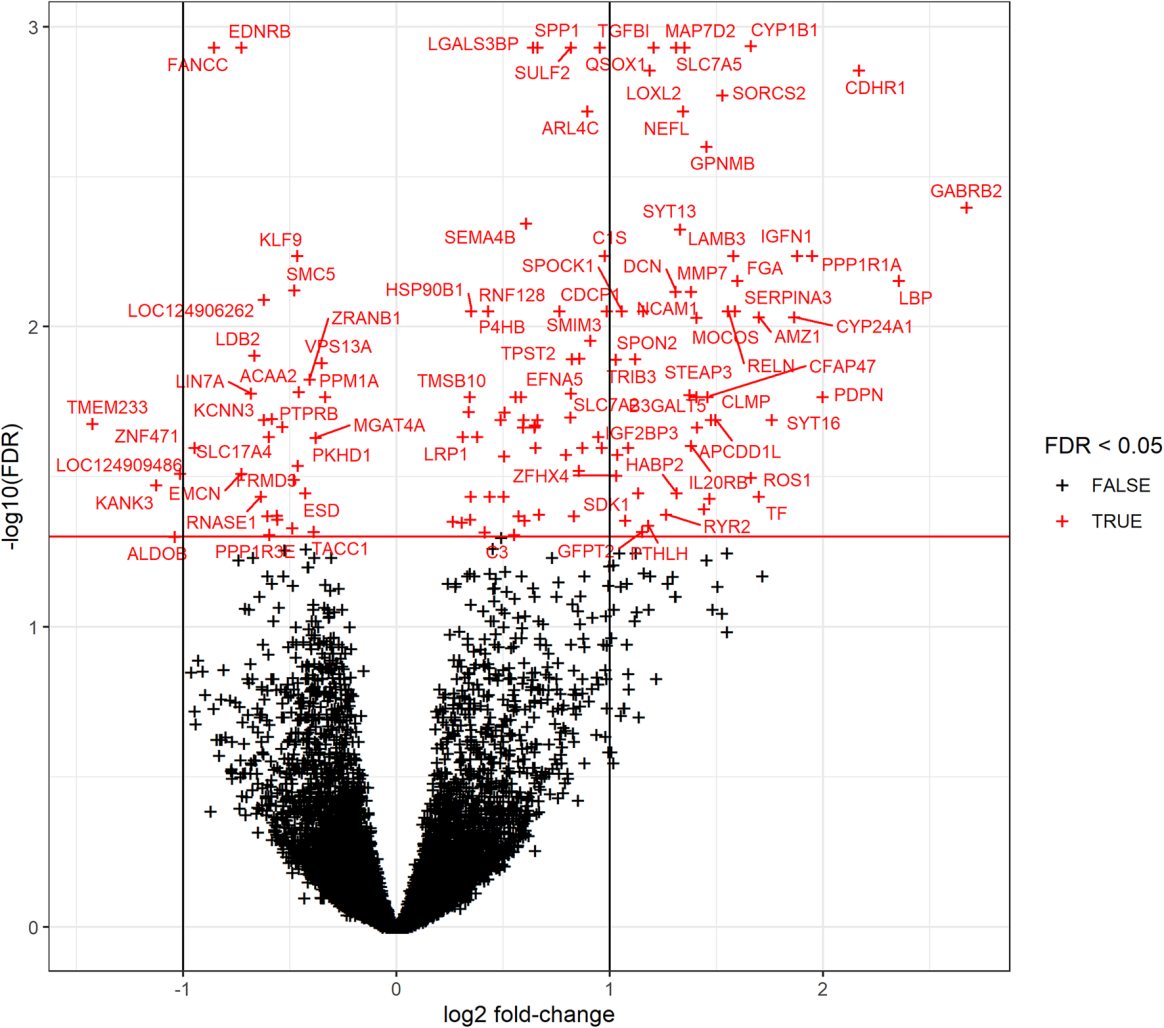
Volcano plot of differentially expressed gene (DEG) analysis. The red horizontal line represents a false discovery rate (FDR) level of 0.05. Genes with a log2 fold-change > 0 were overexpressed, and those with values < 0 were under-expressed.

DEG analysis identified 129 differentially expressed genes between the hyaluronan-positive and hyaluronan-negative groups (Fig. 2). The full DEG list can be found in Supplementary Table S1. Of these genes, 97 were upregulated and 32 were downregulated in the hyaluronan-positive group compared with those in the hyaluronan-negative group. Only protein-coding genes were included, and the FDR was set at < 0.05. There were 53 genes with |log2 fold-change|≥ 1.

### Functional analysis of DEGs

**Figure 3:**
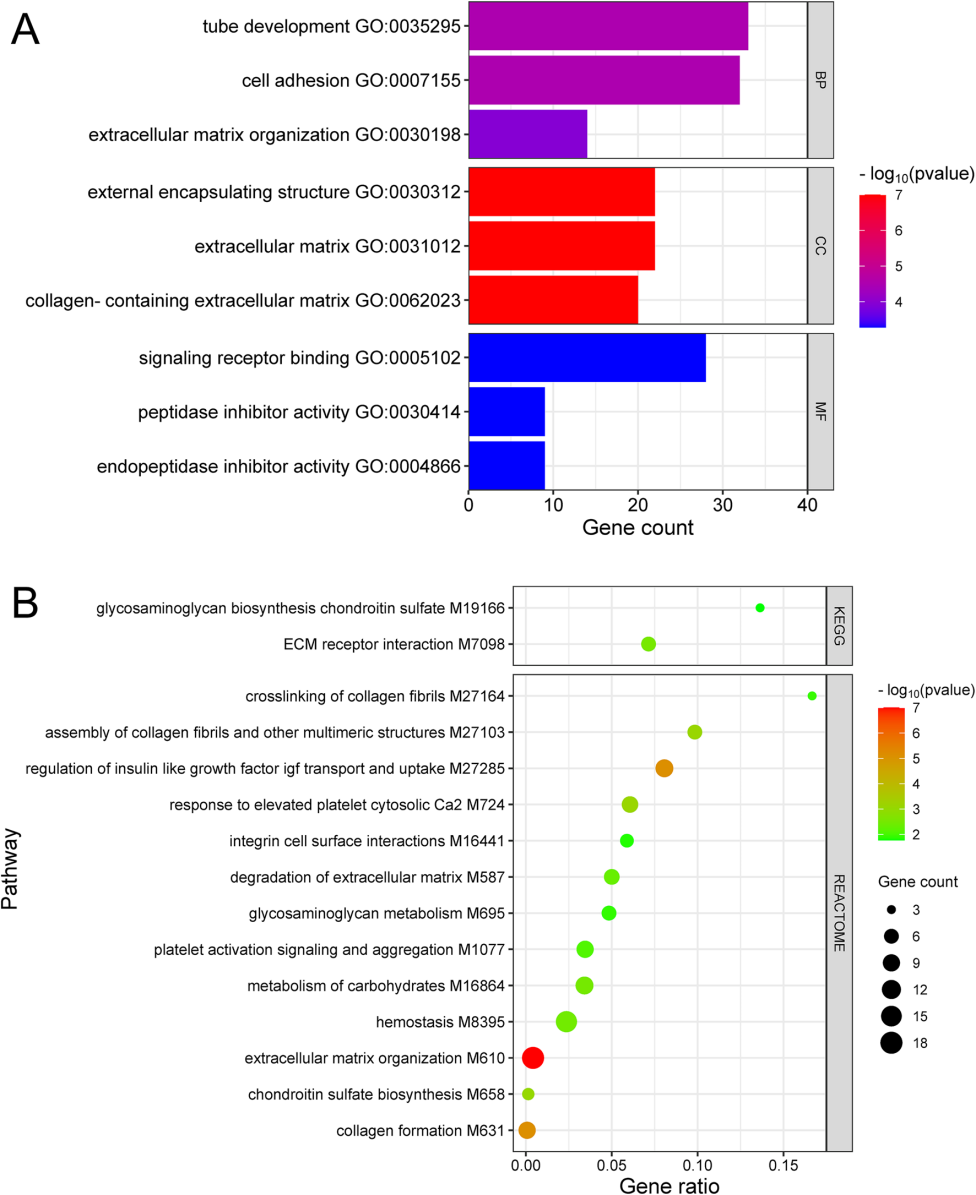
(**A**) ToppGene results of Gene Ontology (GO) and pathway analyses. The bars show the top three most significantly enriched GO terms from each subontology. (**B**) Bubble plot showing the 15 most significantly enriched pathways in the KEGG and REACTOME pathways. *BP* biological process, *CC* cellular compartment, *MF* molecular function, *KEGG* Kyoto Encyclopedia of Genes and Genomes.

The ToppGene portal was used to perform GO, KEGG, and REACTOME enrichment analyses on the biological functions and pathways of the 129 DEGs identified. Gene ontology (GO) analysis revealed that these genes were enriched in 82 biological processes. Of these “tube development”, “cell adhesion”, and “extracellular matrix organization” were the most statistically significant. Of the 31 statistically significant cellular compartments (CC), “the external encapsulating structure”, “extracellular matrix”, and “collagen-containing extracellular matrix” were the most enriched. The most enriched molecular functions (N = 21) were “signaling receptor binding”, “peptidase inhibitor activity”, and “endopeptidase inhibitor activity” (Fig. 3). In terms of KEGG enrichment analysis, three pathways, “ECM receptor interaction” (FDR = 3.07E − 4), “glycosaminoglycan biosynthesis chondroitin sulfate” (FDR = 1.72E − 3) and “complement and coagulation cascades” (FDR = 4.0E − 2), were statistically significantly enriched. In the REACTOME pathway analysis, the top three enriched pathways were “extracellular matrix organization” (FDR = 1.36E − 8), “collagen formation” (p = 7.68E − 6), and “regulation of insulin-like growth factor transport and uptake by insulin-like growth factor-binding proteins” (7.77E − 6) (Fig. 3).

### Gene set enrichment analysis of DEGs

**Figure 4:**
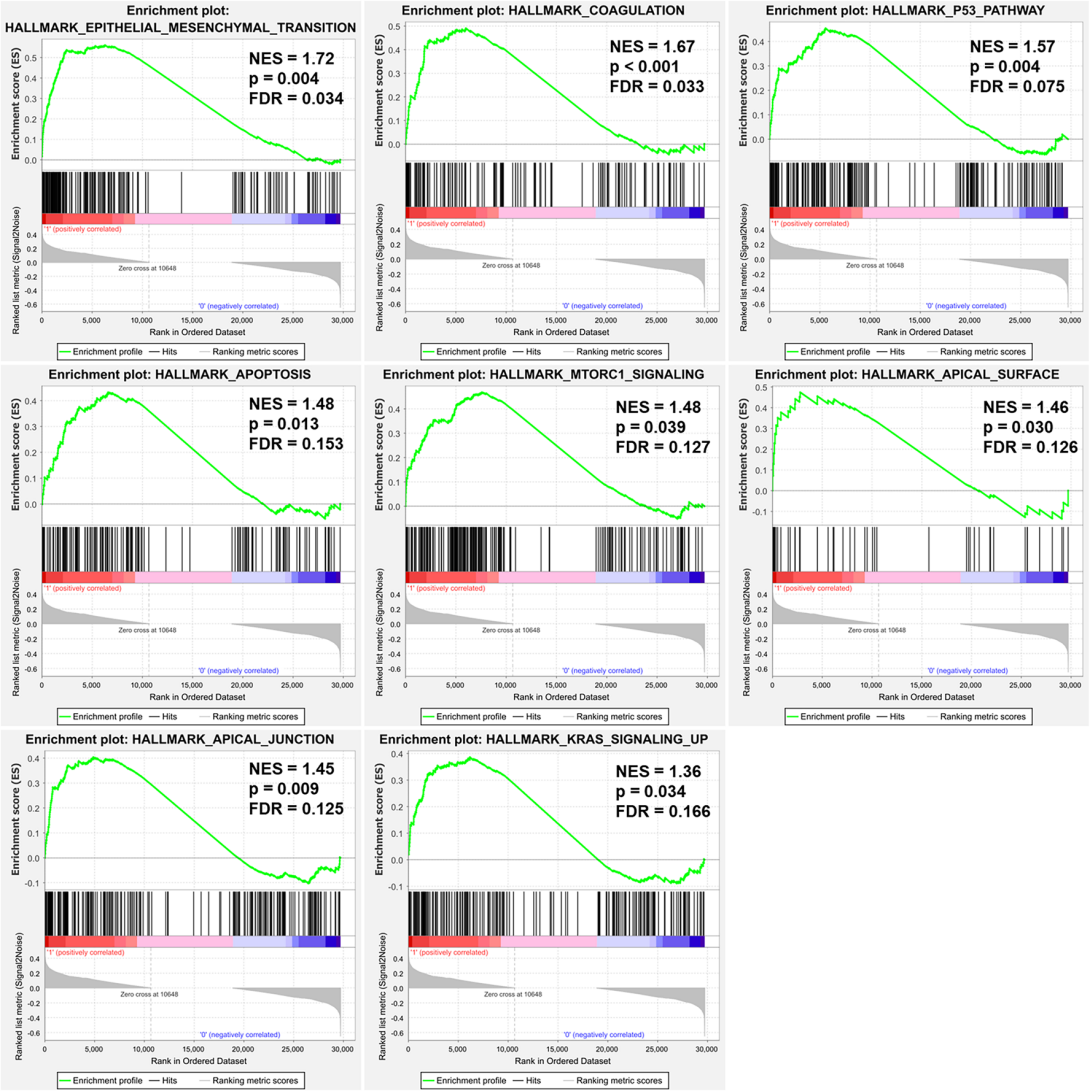
Enrichment plots of Gene Set Enrichment Analysis (GSEA) results of the comparison between HA-positive and HA-negative groups, using the hallmark gene set. *NES* normalized enrichment score, *FDR* false discovery rate.

GSEA analysis showed that 8 gene sets were significantly enriched in the hyaluronan-positive phenotype at FDR < 0.25 and nominal p-value < 0.05. Four gene sets were significantly enriched at FDR < 0.25 and nominal p-value < 0.01. No gene sets were significantly enriched in the hyaluronan-negative phenotype. The pathways with the highest normalized enrichment scores (NES) in the hyaluronan-positive phenotype were epithelial-mesenchymal transition (NES 1.72, FDR = 0.034), coagulation (NES 1.67, FDR = 0.033), P53 pathway (NES 1.57, FDR = 0.075), apoptosis (NES 1.48, FDR = 0.153), MTORC1 signaling (NES 1.48, FDR = 0.127), apical surface (NES 1.46, FDR = 0.126), apical junction (NES 1.45, FDR = 0.125), and KRAS signaling up (NES 1.36, FDR = 0.166). The enrichment plots are shown in Fig. 4.

### Protein-to-protein interaction network and subnetworks

A PPI network was constructed from 129 DEGs, using a minimum interaction score of 0.4. The network contained 127 nodes and 172 edges (PPI enrichment, p < 1.0E − 16). Fifty of the nodes were singletons with no connection to other nodes. The PPI network is shown in Supplementary Figure S1. The MCODE plug-in identified five closely connected subnetworks from the PPI network; the highly connected regions are shown in Supplementary Table S2. These networks contained 27 unique genes. ToppGene GO revealed that these genes were mostly associated with the molecular functions “endopeptidase inhibitor activity” and “peptidase inhibitor activity”, the biological processes “locomotion and cartilage development”, and the cellular components “extracellular matrix and external encapsulating structure”. The most enriched REACTOME pathway was “extracellular matrix organization” and the most enriched KEGG pathway was “glycosaminoglycan biosynthesis-chondroitin sulfate”.

### Hub gene discovery and analysis

**Figure 5:**
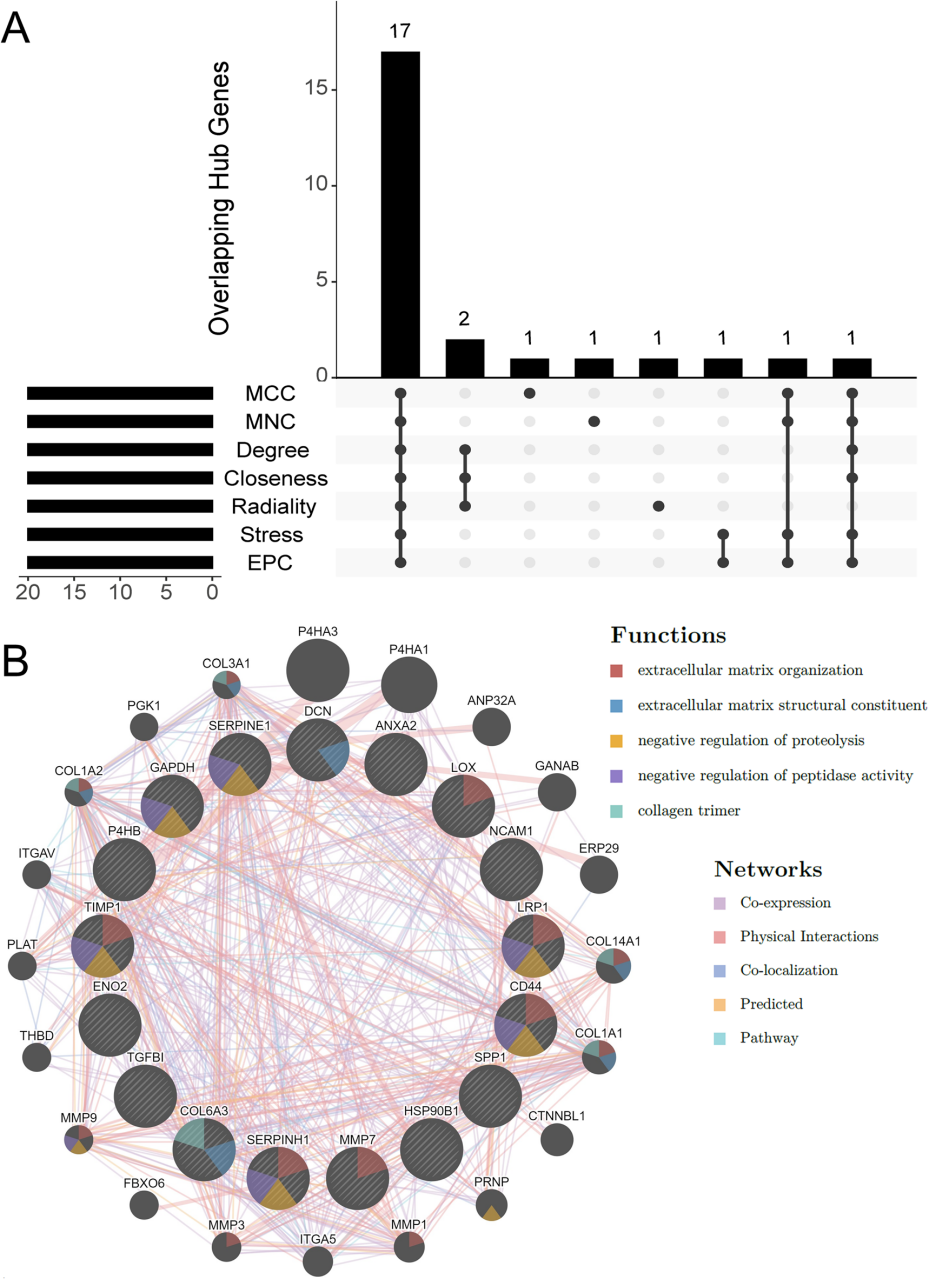
Overlapping hub genes and co-expression networks of hub genes. (**A**) UpSet intersection plot showing the seven algorithms used to identify 17 overlapping hub genes. (**B**) Hub genes (inner circles) and 20 co-expressed genes. Circle diameter correspond to score assigned to each gene (GeneMANIA).

Using seven ranking algorithms of Cytoscape’s CytoHubba plug-in, we calculated the top 20 hub genes (Supplementary Table S3). The intersection of these results revealed 17 common hub genes: *ANXA2*, *CD44*, *COL6A3*, *DCN*, *ENO2*, *GAPDH*, *HSP90B1*, *LOX*, *LRP1*, *MMP7*, *NCAM1*, *P4HB*, *SERPINE1*, *SERPINH1*, *SPP1*, *TGFBI*, and *TIMP1* (Fig. 5A). The full names and functions of the genes are listed in Table 2. All the common hub genes were overexpressed in HA-positive tumors compared with HA-negative tumors. The most enriched GO ontologies, as well as the KEGG and REACTOME pathways, did not differ significantly from those of the DEGs (Supplementary Dataset).

**Table 2:** 17 hub genes.

Gene symbol	Gene function	References
*ANXA2*	ANXA2 is a calcium-dependent phospholipid-binding protein expressed mainly on cell membranes, and it participates in many cellular functions, such as exocytosis, endocytosis, and redox regulation. ANXA2 has been found to promote cell migration and mobility, and its overexpression indicates poorer prognosis in RCC	^66,67^
*CD44*	A non-kinase cell surface transmembrane glycoprotein with variant isoforms. It is involved in cell–cell interaction and promotes cell adhesion and migration. It binds many ligands, such as hyaluronic acid and matrix metalloproteinases and has been widely implicated as a cancer stem cell marker in several cancers. Overexpression of CD44 has been associated with stem-like features and epithelial-mesenchymal transition. Higher expression predicted poorer prognosis in RCC	^68,69^
*COL6A3*	Collagen type VI alpha 3 chain *(COL6A3*) encodes one of three chains constituting type 6 collagen (COL6), which is an extracellular-matrix protein. In cancer, COL6 is involved in the regulation of apoptosis, autophagy, fibrosis, angiogenesis, and inflammation. Higher expression of COL6A3 relates to poor prognosis and occurs in metastatic ccRCC	^70,71^
*DCN*	Decorin *(DCN)* encodes a member of the small leucine-rich proteoglycan family of proteins. Protein product is present in extracellular matrix and conveys antitumorigenic properties by binding collagen and multiple growth factors. DCN has been identified as a tumor suppressor in RCC	^72,73^
*ENO2*	Enolase2 is a homodimer that regulates glycolysis and is widely involved in other pathophysiological processes of different malignancies. ccRCC patients with ENO2 overexpression have worse clinical features and prognosis	^74,75^
*GAPDH*	Glyceraldehyde-3-phosphate dehydrogenase (GAPDH) is a vital enzyme for energy metabolism as a regulator of the glycolytic cascade, including anaerobic glycolysis. Recent studies have also shown its association with tumorigenesis, apoptosis, and cell proliferation. Higher expression levels of GAPDH are associated with worse prognosis in ccRCC patients	^76,77^
*HSP90B1*	Heat shock protein 90 beta family member 1 (HSP90B1) is a member of the heat-shock protein 90 family. It maintains endoplasmic reticulum (ER) stress sensors and preserves ER protein folding capacity. It has been linked to immune response and cancer development. Expression of HBP90B1 was found to be higher in and to convey poorer prognosis in RCC	^78^
*LOX*	Lysyl oxidase (LOX) is a member of the lysyl oxidase family of proteins. They catalyze oxidative deamination of lysine and hydroxylysine to form allysine, the first step in the collagen cross-linking reaction. In addition, these enzymes act as transcription factors promoting epithelial-mesenchymal transition. LOX has been found to be upregulated in ccRCC and to convey poorer prognosis	^79–81^
*LRP1*	LDL receptor related protein 1 (*LRP1*) encodes a member of the low-density lipoprotein receptor family. It is involved in endocytosis and the regulation of signaling pathways. Its overexpression has been associated with tumor cell migration and invasion. Higher LRP1 expression has been identified in RCC compared with normal kidney and higher expression conveys poorer prognosis	^82,83^
*MMP7*	Matrix metallopeptidase 7 (MMP7) is a member of the matrix metalloproteinase family, a group of 23 zinc-dependent endopeptidases. MMP7 degrades various extracellular matrix substrates and plays a role in wound healing, bone growth, and inflammation. MMP7 is expressed in many types of cancer cells, and it promotes tumor progression by inhibiting apoptosis. Higher expression in ccRCC has been associated with worse prognosis	^84,85^
*NCAM1*	Neural cell adhesion molecule 1 (NCAM1), also known as CD56, is a member of the immunoglobulin superfamily found in cells of neural lineage and hematopoietic cells. The protein product is involved in cell-to-cell and well as cell–matrix interactions during development and differentiation. In RCC protein expression has been associated with higher metastatic potential and poorer prognosis	^86,87^
*P4HB*	The protein product of this gene is the beta subunit of prolyl 4-hydroxylase, which is a multifunctional enzyme belonging to protein disulfide isomerase-family. The protein functions as a chaperone and prevents protein misfolding. It has been shown to promote progression of malignant tumor, including RCC, where it is associated with poor prognosis	^88,89^
*SERPINE1*	Serpin family E member 1 (SERPINE1) is an inhibitor of urokinase and tissue plasminogen activator. It has been shown to play roles in cell adhesion, migration, invasion, and tumor vascularization. It is associated with poorer prognosis in various cancers, including ccRCC	^86,90^
*SERPINH1*	Serpin family H member 1 (*SERPINH1*) encodes protein HSP47, which is an important chaperone required for the correct folding and secretion of collagen. HSP47 promotes tumor growth and invasion in many malignancies, such as cervical, pancreatic, and breast cancers, probably by modifying ECM. Increased SERPINH1 expression has been associated with worse prognosis in RCC	^91,92^
*SPP1*	Secreted phosphoprotein 1 (SSP1) is a member of the small integrin-binding ligand N-linked glycoprotein family of proteins, which bind and activate matrix metalloproteinases in cancer. It functions in immune response, biomineralization, and tissue remodeling. It has been proven to be overexpressed in various cancers, such as ovarian cancer, glioblastoma, hepatocellular carcinoma, and prostate cancer. In renal cell carcinoma, SPP1 indicated poor prognosis	^93,94^
*TIMP1*	TIMP metallopeptidase inhibitor 1 (TIMP1) is a natural inhibitor of matrix metalloproteinases. TIMPs participate in biological processes of anti-apoptosis, anti-angiogenesis, cell cycle regulation, and differentiation. TIMP1 is significantly upregulated in cell lines and RCC tissues. Higher expression of TIMP1 indicated a poor prognosis	^95^
*TGFBI*	Transforming growth factor beta induced (TGFBI) localizes in extracellular matrix and contributes to cell-collagen interactions and bone formation. TGFBI has been reported to have both tumor promoting and suppressing roles. Higher TGFBI expression levels have been shown to predispose to worse survival in ccRCC	^96,97^

Next, we analyzed the common hub genes using the GeneMANIA database. The input gene list generated a composite network, with network weights for individual subnetworks of 51.05% for co-expression, 42.77% for physical interactions, 3.51% for colocalization, 2.64% for predicted, and 0.04% for pathways (Fig. 5B). GO functional enrichment analysis showed that nine of the 20 co-expressed genes were enriched in the biological processes of “extracellular matrix organization”, “extracellular structure organization”, and “external encapsulating structure organization”. Furthermore, 11/20 genes were involved in the REACTOME “extracellular matrix organization” pathway and 8/20 in “collagen formation” (Supplementary Dataset).

Finally, TFs that regulate hub genes were predicted using the TRRUST database. Eleven candidate regulators were identified, including *CEBPA*, *CTNNB1*, *HDAC1*, *HIF1A*, *JUN*, *NFKB1*, *RELA*, *RUNX1*, *SP1*, *STAT3* and *TWIST1* (Supplementary Table S4). We used publicly available TCGA-KIRC data to analyze the expression levels of these TFs in normal renal tissue as well as in inferred “HA-negative” and “HA-positive” phenotype TCGA samples (Supplementary Fig. S2). The analysis showed that *TWIST1*, *RUNX1*, *CEBPA*, and *RELA* were upregulated in carcinoma samples compared with normal renal tissues, whereas *HIF1A* was downregulated. The HA-positive phenotype had statistically higher expression of *TWIST1*, *RUNX1*, and *CEBPA* than did the HA-negative phenotype. Conversely, *CTNNB1*, *HDAC1*, *RELA1*, *NFKB1*, and *STAT3* were downregulated in the HA-positive phenotype.

### Signatures of differentially expressed genes identify prognostic groups in the TCGA-KIRC cohort

**Figure 6:**
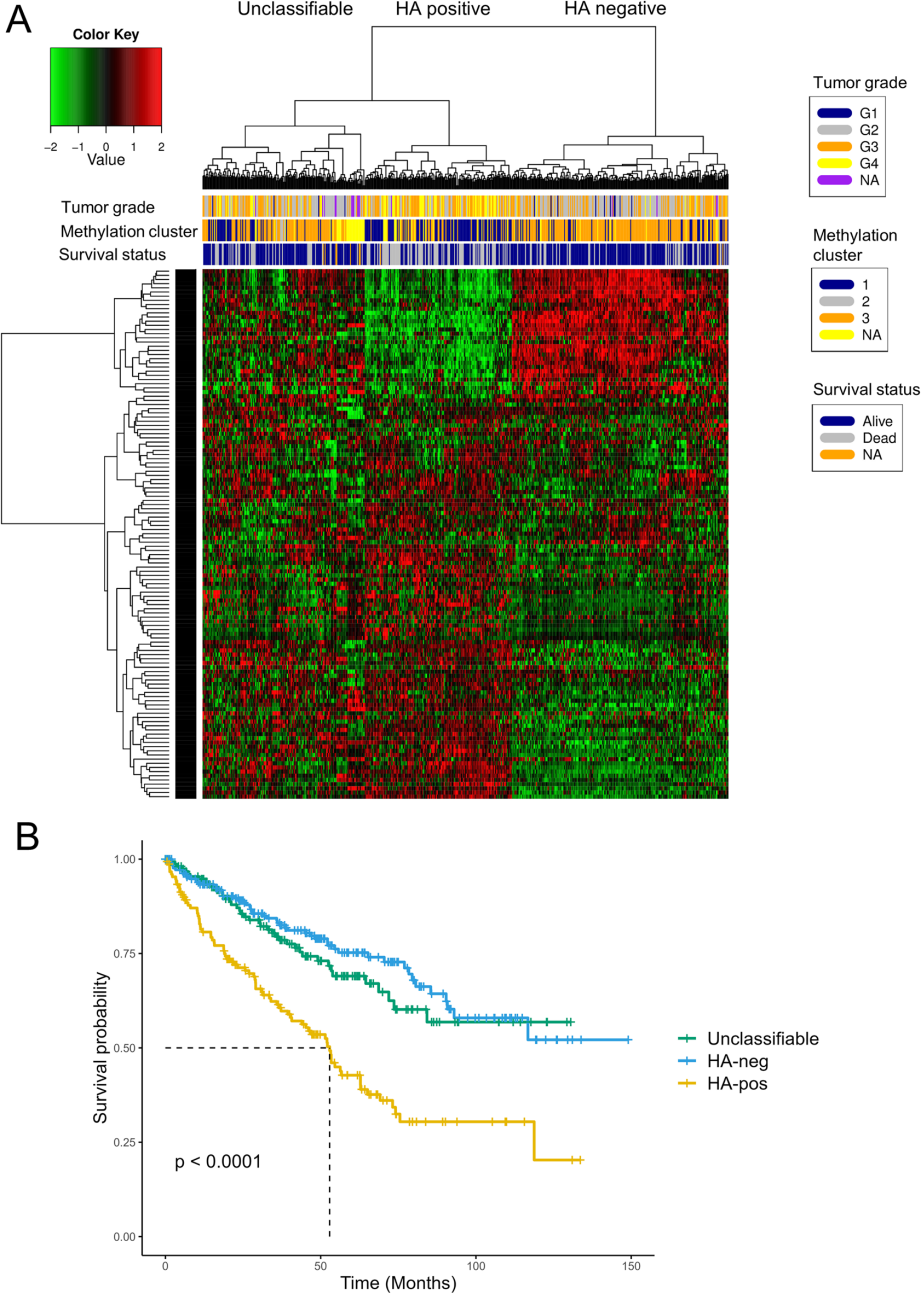
(**A**) Heatmap and cluster analysis of TCGA data using a set of 129 differentially expressed genes (NOt Just Another Heatmap). The color key denotes log2 fold-change. (**B**) Survival plot of TCGA heatmap clusters. Log-rank test is used to calculate p-value.

To explore our findings, we subjected TCGA-KIRC data to heatmapping and cluster analysis using the previously identified DEG set. The NOJAH tool was used for analysis. Heatmap plotting and cluster analysis revealed three distinct groups (Fig. 6A). A total of 151 samples had a gene expression profile similar to that of the HA-positive sequencing group, hereafter referred to as the HA-positive cluster. A total of 221 samples had a gene expression profile similar to that of the hyaluronan-negative sequencing group, hereafter referred to as the HA-negative cluster. The remaining samples (n = 165) were unclassifiable, based on our set of DEGs. Information about survival status, tumor pathological stage and tumor grade were available for each sample, and the hyaluronan-positive cluster was associated with death (Pearson chi-squared test, p < 0.001), higher tumor grade (Pearson chi-squared test, p < 0.001), higher stage (Pearson chi-squared test, p < 0.001) and methylation cluster 1 (Pearson chi-squared test p < 0.001). Grade 4 tumors tended to cluster in the HA-positive cluster. Comparison of the clinical characteristics between discovery set and TCGA clusters are shown in Supplementary Table S5. The Kaplan–Meier estimator showed worse prognosis for the HA-positive cluster in the TCGA data. There was no significant difference between the unclassifiable and HA-negative clusters (Fig. 6B).

### TCGA GSEA analysis

GSEA revealed that 7 gene sets were significantly enriched in the HA-positive cluster at FDR < 0.25 and nominal p-value < 0.05. Two gene sets with similar confidence scores were enriched in the HA-negative cluster. The pathways with the highest positive NES values were IL6 JAK STAT3 signaling (NES 2.01, FDR = 0.066), epithelial-mesenchymal transition (NES 1.98, FDR = 0.047), inflammatory response (NES 1.97, FDR = 0.036), allograft rejection (NES 1.92, FDR 0.045), glycolysis (NES 1.68, FDR = 0.188), coagulation (NES 1.63, FDR = 0.205), and estrogen response late (NES 1.57, FDR = 0.177). The pathways with the lowest NES values were fatty acid metabolism (NES − 1.72, FDR 0.334) and β-catenin signaling (NES − 1.66, FDR 0.240). All pathways are available in the Supplementary Dataset.

### Hub gene expression analysis

To analyze the hub gene expression pattern, we examined the relative mRNA expression levels in the TCGA-KIRC data. We compared the expression levels between the two phenotypes of HA-positive and HA-negative clusters that we identified in the heatmapping and cluster analysis. In addition, the mRNA expression levels of normal renal tissues were included in the analysis. The results showed that the following genes were significantly overexpressed in renal cell carcinomas with respect to normal renal tissue: *ANXA2*, *CD44*, *COL6A3*, *ENO2*, *GAPDH*, *HSP90B1*, *LOX*, *LRP1*, *NCAM1*, *P4HB*, *SERPINE1*, *SERPINH1*, *TGFBI* and *TIMP1. SPP1* and *DCN* were significantly downregulated in carcinomas in comparison with normal renal tissue. All hub genes were overexpressed in the HA-positive cluster, consistent with our sequencing results (Supplementary Fig. S3).

### Prognostic value of hub genes

Hub genes were subjected to survival analysis by KMplotter using the TCGA-KIRC data. This indicated that high expression levels of *ANXA2*, *CD44*, *COL6A3*, *DCN*, *ENO2*, *GAPDH*, *MMP7*, *P4HB*, *SERPINH1*, *TGFBI*, and *TIMP1* were associated with unfavorable overall survival. No statistical difference in overall survival was observed with different expression levels of *HSP90B1*, *LOX*, *LRP1*, *NCAM1*, *SERPINE1*, and *SPP1* (Supplementary Fig. S4 and S5). However, when subjected to disease-free survival (DFS) analysis, only the high expression levels of *TGFBI* and *P4HB* were statistically significant (Supplementary Fig. S6 and S7).

## Discussion

In this study, we examined RNA expression levels in hyaluronan-positive and-negative tumors. To the best of our knowledge, this is the first study where tumoral hyaluronan accumulation has been studied through RNA sequencing. To date, hyaluronan has been studied in renal cell carcinoma in terms of individual protein and mRNA levels. However, the molecular pathways associated with hyaluronan accumulation have remained unclear.

DEG analysis revealed 129 genes with expression profiles that differed between the HA-positive and HA-negative cohorts. In total, 97 genes were up-regulated in the HA-positive group. These DEGs appeared to regulate pathways involved in the extracellular matrix, collagen formation, carbohydrate metabolism, and cellular adhesion. The most enriched gene ontology terms were related to “peptidase inhibitor activity”, “cell adhesion”, and “collagen-containing extracellular matrix”. GSEA revealed that the DEGs were enriched in similar pathways, such as the extracellular matrix, apical surface, and apical junction. These results are in line with previous findings that hyaluronan accumulation is associated with epithelial-mesenchymal transition, cell adhesion, and extracellular matrix organization^7,98–101^.

Among the DEGs, the hyaluronan-associated molecules CD44 and HABP2 were identified. CD44 is a hyaladherin, and HA-CD44 interaction has been shown to promote tumor cell survival and chemoresistance^102^. HABP2, on the other hand, is a serine protease that promotes migration, extravasation, tumor growth, and metastasis in lung cancer^103^. This protein also has a peculiar feature in that its expression is increased by low-molecular-weight HA (LMW-HA) and decreased by high-molecular-weight HA (HMW-HA)^104^. The absence of other hyaluronan-binding proteins from the DEG list, as well as hyaluronan-synthesizing and hyaluronan-degrading enzymes, is an interesting finding. This might reflect the previous observations that transcriptional regulation may not be the main driver of altered HA levels in RCC^105^. Therefore, alternative mechanisms (e.g., those related to the supply of HA substrates or regulation of translational and enzymatic activity) may explain HA accumulation in these tumors. RCC is a metabolically active disease, and tumors have been shown to increase the uptake and utilization of glucose and produce increased amount of pentose phosphate pathway (PPP) intermediates^106^. UDP-sugars, a type of PPP intermediate necessary for HA synthesis, have been shown to accumulate in breast carcinomas and strongly correlate with tumor HA levels independent of the mRNA levels of HA synthases^107^.

KEGG pathway “glycosaminoglycan biosynthesis chondroitin sulfate” and REACTOME pathway “glycosaminoglycan metabolism” were enriched in HA-positive tumors. This was mainly due to the overexpression of *CHST11*, *DCN*, *CHPF*, and *CHST3*, which are common to both pathways. These genes participate in the sulfation and biosynthesis of chondroitin sulfate (CS) as well as its organization in extracellular matrix^108,109^. CS has been observed to be elevated in breast cancer stroma, and increased CS levels are associated with poor differentiation status in hepatocellular carcinoma and in advanced stage and recurrent ovarian cancer^110–112^. Furthermore, there is significantly more CS in RCC tissues, and the CS biosynthesis pathway is upregulated in RCC compared with non-neoplastic kidney tissues^113,114^. The relationship between CS biosynthesis and HA accumulation in ccRCC remains unclear.

Gene set enrichment analysis showed that the mTORC1 signaling gene set was enriched in HA-positive tumors. Mechanistic target of rapamycin (mTOR) is a serine/threonine kinase involved in cellular growth, proliferation, and autophagy. mTOR activation plays a major role in RCC, and mTOR inhibitors have been used to treat metastatic RCC. Regrettably, mTOR inhibitors have low objective response rates, and tumors rapidly develop resistance^115^. Nevertheless, some patients with mTOR signaling-activating genomic alterations show long-lasting responses^116^. Therefore, tumors with HA accumulation may respond more favorably to mTOR inhibition.

Hub gene analysis identified 17 highly connected genes. These genes were involved in the same molecular pathways as DEGs, and 11 of them (*ANXA2*, *CD44*, *COL6A3*, *DCN*, *ENO2*, *GAPDH*, *MMP7*, *P4HB*, *SERPINH1*, *TGFBI*, and *TIMP1*) were correlated with poor prognosis in renal cell carcinoma at the mRNA level when the median cut-off was used. Many of the proteins encoded by these genes function in cell adhesion (CD44, TGFBI, COL6A3, SPP1, and NCAM1), participate in glycolysis (GAPDH and ENO2), exhibit protease activity (P4HB, HSP90B1, and TIMP1), and act as chaperones (SERPINE1 and SERPINH1) (Table 2). These proteins, besides CD44, have little to no known interaction with hyaluronan. However, LRP1 (a low-density lipoprotein receptor involved in endocytosis and regulation of signaling pathways) has been shown to interact with artificial sulfated hyaluronan in bone regeneration studies^117^. The protein expression of CD44 has been studied in renal cell carcinoma, and higher expression is associated with poor prognosis^118^. In addition, high protein expression of ANXA2, ENO2, P4HB, SERPINH1, TGFBI, and LRP1 is associated with poor prognosis in renal cell carcinoma^67,89,92,119–121^. Patraki and Cardille^122^ showed that MMP7 was more strongly expressed in high-grade RCC. However, no survival analysis was conducted. Furthermore, COL6A3 expression was shown to be higher in ccRCC metastases than in primary tumors^70^. Not surprisingly, the top 20 related genes identified by co-expression analysis were associated with the same biological processes as the hub genes. Hub genes and their co-expressing genes act as potential targets for downstream analyses.

Transcription factor analysis revealed that 11 genes were associated with our set of hub genes. Of notable interest is *RUNX1*, which is highly expressed in renal cell carcinoma compared with normal kidney tissue. Furthermore, *RUNX1* expression was higher in the HA-positive cluster. *RUNX1* has been shown to affect multiple biological processes, such as proliferation, apoptosis and differentiation, and lineage determination. In addition, its involvement as a fusion partner in acute myeloid leukemia (AML) is well-known^123^. Recently, it has been associated with other malignancies, such as promoting EMT in colorectal carcinoma by activating the Wnt/β-catenin signaling pathway^124^. *RUNX1* has previously been associated with poor prognosis in renal cell carcinoma^125^. Moreover, Rooney et al.^126^ showed that deletion of *RUNX1* in ccRCC cell lines reduced tumor cell growth and viability. *RUNX1* deletion caused many alterations to biological pathways, notably cell adhesion and ECM modelling. Interestingly, the second most altered gene ontology observed was “eph and ephrin signaling”, which is a downstream target of the WNT signaling pathway. One of the genes with the most significantly altered expression was *SERPINH1*, one of the hub genes identified. Deletion of *RUNX1* caused a significant reduction in SERPINH1 levels, indicating that RUNX1 is a positive regulator of SERPINH1. Interestingly, SERPINH1 has been shown to regulate EMT through Wnt/β-catenin signaling in gastric cancer^127^. The interplay between RUNX1, SERPINH1, and hyaluronan could offer insights into the molecular mechanisms underlying the hyaluronan-induced migratory phenotype and EMT.

To investigate our previous and present findings, we performed in silico heatmap clustering using the list of DEGs and TCGA-KIRC data^14^. Heatmap clustering identified two prognostically divergent clusters whose gene expression patterns corresponded to those of our cohorts. A gene expression pattern similar to that of our HA-positive sequencing group conveyed a poorer prognosis in the TCGA data. This cluster was enriched in tumors with a higher tumor grade and pathological stage. Additionally, it exhibited a methylation cluster that has previously been shown to be associated with the CpG island methylator (CIMP) phenotype and increased Wnt signaling pathway activity^63^. Motzer er al.^128^ molecularly categorized ccRCC into seven subtypes, using an integrated multi-omics approach. Two subtypes (clusters 1 and 6) were enriched with stromal transcriptional signatures, and cluster 6 contained a substantial proportion of sarcomatoid ccRCCs. Interestingly, cluster 1 showed enrichment of WNT signaling genes, in addition to high expression of genes related to the TGF-β, Hedgehog, and NOTCH signaling pathways. Regrettably, TCGA clinical data did not contain information regarding possible sarcomatoid changes. However, it could be deduced that since most of the grade 4 tumors were clustered in the HA-positive cluster, some of these were sarcomatoid. This is in line with our previous finding that HA accumulates in sarcomatoid carcinomas^14^.

In silico GSEA analysis of TCGA data revealed that genes in the HA-positive cluster were enriched in pathways associated with inflammation. These included “IL6/JAK/STAT3-signaling”, “inflammatory response”, and “allograft rejection”. It has been previously shown that hyaluronan-rich stroma and low-molecular-weight hyaluronan (LMW-HA) promote inflammation and cytokine production^129,130^. Kainulainen et al.^131^ showed that upregulation of proinflammatory genes in MV3 melanoma cells stimulated synthesis of a peritumoral HA coat. In addition, an increased number of HA-containing HYAL2^+^PD-L1^+^ myeloid-derived suppressor cells (MDSCs) have been observed in ccRCC, promoting HA degradation to LMW-HA, cancer-related inflammation, and immunosuppression^132^. Furthermore, HYAL2^+^ myeloid cells have been associated with HA degradation and angiogenesis in bladder cancer^133^. There is evidence that HA can modulate immune cell infiltration in the tumor microenvironment by binding to, polarizing, and recruiting macrophages. It is also well appreciated that immunity and angiogenesis are closely interlinked^134,135^. As RCC is one of the most immune-infiltrated tumors, it may be possible to improve the efficacy of cancer treatments, such as immunotherapy, by targeting HA^25,136,137^.

Our study had some limitations. The study design involving samples collected in the period 2000–2013 and their FFPE nature likely diminished the sensitivity and increased the variation. However, analyzing three punch cores from each tumor should compensate for the random scatter. Due to the inherent degradation of RNA, the count values were generally low, as expected. This phenomenon may have led to limited coverage of the full transcriptomic differences between the groups and posed limitation to the effective cross-validation of the dataset. In addition, the lack of independent known-label datasets with respect to hyaluronan status posed a hindrance, preventing us from leveraging such datasets to validate our results. Finally, owing to the relatively low number of underexpressed genes, GSEA analyses did not necessarily have sufficient statistical power to identify underexpressed gene sets.

## Conclusions

Our study demonstrates that hyaluronan accumulation is associated with biological pathways related to the extracellular matrix, EMT, and cell–stroma interactions. The gene expression signature we discovered was associated with poor prognosis and a higher tumor grade in ccRCC. Whether the pathways identified in this study lead to hyaluronan accumulation or whether HA accumulation induces certain genes remains unclear. Identification of these pathways may open new avenues for hyaluronan research in renal cell carcinomas and other human malignancies. Further studies involving independent in silico and wet lab validation sets are required to validate these results.

### Supplementary Information

Supplementary Information 1.

Supplementary Information 2.

## Data Availability

The data that support the findings of this study are available from the Biobank of Eastern Finland, but restrictions apply to the availability of these data, which were used under license for the current study and so are not publicly available. However, the data are available from the authors upon reasonable request and with permission from the Biobank of Eastern Finland. Please contact info@ita-suomenbiopankki.fi for access inquiries.
