# Supplementary material for: Differential expression analysis identifies a prognostically significant extracellular matrix–enriched gene signature in hyaluronan-positive clear cell renal cell carcinoma
Source: Sci Rep. 2024 May 9;14:10626. doi: 10.1038/s41598-024-61426-3 (PMC11082176; doi:10.1038/s41598-024-61426-3)
Supplement: Supplementary file 2 — Supplementary Information 2. [file 41598_2024_61426_MOESM2_ESM.pdf]

Differential expression analysis identifies a prognostically significant and extracellular matrix–enriched gene signature in hyaluronan-positive clear cell renal cell carcinoma

Otto Jokelainen MD, Teemu Rintala MSc, Vittorio Fortino PhD, Sanna Pasonen-Seppänen PhD, Reijo Sironen MD, PhD, Timo K. Nykopp MD, PhD

Supplementary Table S1. List of differentially expressed genes (FDR < 0.05).

Gene Symbol	log2 fold-change	FDR
GABRB2	2.67245007713722	0.00399148825721624
LBP	2.35609042976598	0.00699701401405195
CDHR1	2.16932485280254	0.001396498006425
PDPN	1.99796484464068	0.0170777130178846
PPP1R1A	1.94835180828737	0.00578162094102943
IGFN1	1.87782108423335	0.00578939666591253
CYP24A1	1.86512023847026	0.00926308888351784
SYT16	1.75977314051748	0.0203567870968191
AMZ1	1.69926746943328	0.00926308888351784
TF	1.69872516503333	0.0366932180402097
ROS1	1.66161209147931	0.0318110024916133
CYP1B1	1.6613664073866	0.00115715596274392
FGA	1.59849389577354	0.00699701401405195
SERPINA3	1.58729795653353	0.00884449446301598
LAMB3	1.58054864573659	0.00578162094102943
RELN	1.55500620340653	0.00886257554647448
SORCS2	1.5292312209273	0.00169049366464666
APCDD1L	1.49679632862449	0.0203567870968191
CLMP	1.47510000774276	0.0203567870968191
SCNN1G	1.46707161173871	0.0372167303116845
CFAP47	1.45899625960386	0.0170777130178846
GPNMB	1.45398763493901	0.00250108711922886
HS3ST3A1	1.44082199939277	0.0404643688353786
IGF2BP3	1.40872833343328	0.0215406974448497
MOCOS	1.40667506156473	0.00930880802698873
B3GALT5	1.40589817477605	0.0170777130178846
MMP7	1.38194279686624	0.00761500849594177
IL20RB	1.38063036580441	0.024812514408566
STEAP3	1.37452634571657	0.0168180265140396
MAP7D2	1.35022050099954	0.00116949773452006
NEFL	1.34468049517644	0.0019071423545221
SYT13	1.32924148775256	0.00472896015925684
HABP2	1.31371917100673	0.0357557074858978
SLC7A5	1.31130051498896	0.00116949773452006
DCN	1.30966995105163	0.00761500849594177
RYR2	1.26495911976681	0.0420805241884081
TGFBI	1.20561434865444	0.00116949773452006
LOXL2	1.18835962036135	0.001396498006425
PTHLH	1.18051973788244	0.0457766600661182
NCAM1	1.15806941030107	0.00884449446301598
GFPT2	1.15333462153443	0.047980932585511
SDK1	1.13395125530041	0.0357557074858978
SPON2	1.12034148735572	0.0127510099207857
PDE1A	1.08687038551614	0.0252034728788225
IGDCC4	1.0723376449853	0.0442037197343307
SPOCK1	1.05639636342339	0.00886257554647448
ADAM12	1.03473803319478	0.0265797303643994
ZFHX4	1.03167322388167	0.0312457284089984
TRIB3	1.0295283325606	0.0128329227736126
CDCP1	0.986633787167516	0.00886257554647448
C1S	0.977183017484635	0.00578162094102943
SEMA3C	0.962620325829683	0.0252034728788225
QSOX1	0.954034897837004	0.00116949773452006

NEB	0.946507676156865	0.0232287095088901
SMIM3	0.909151331427928	0.0110953259470109
ARL4C	0.896077217445216	0.0019071423545221
TBC1D2	0.872488352293895	0.0252034728788225
EFNA5	0.85722141512101	0.0127273825747519
CHST3	0.856443805251588	0.0301399103921804
SERPINE1	0.831093708377577	0.0426404033268697
TPST2	0.823399441702152	0.0127510099207857
SULF2	0.81901912136952	0.00116949773452006
SLC7A2	0.818270507692956	0.0166465340970221
PDE10A	0.815580228731175	0.0200077613274264
DOCK11	0.794051508844827	0.0265797303643994
RNF128	0.76471345685614	0.00884449446301598
COL6A3	0.669441759476337	0.0421053192713588
SPP1	0.662406157097244	0.00116949773452006
LOX	0.661080429397365	0.0203567870968191
SYTL2	0.653062926623908	0.0252034728788225
CHST11	0.648887179715858	0.0212799526024911
CDK2AP2	0.64822568769799	0.0215406974448497
LGALS3BP	0.641071722970343	0.00116949773452006
SEMA4B	0.606765974619319	0.00451733539323328
CHPF	0.599944031490276	0.0442037197343307
NPAS2	0.596677428121321	0.0203567870968191
PXDN	0.593571191067053	0.0215406974448497
CD44	0.585335680582554	0.0170777130178846
ELF4	0.57245470904779	0.0426404033268697
ITGA3	0.55861349939465	0.0170777130178846
C3	0.552297048436784	0.0491198828252648
PLOD2	0.506655969121153	0.0192092818354607
SERPINH1	0.504018329036588	0.0269485289032031
MGST1	0.502693794255665	0.0366932180402097
ENO2	0.487609437348325	0.0203375431644869
TIMP1	0.437102928431194	0.0366932180402097
P4HB	0.429484894266236	0.00884449446301598
ANXA2	0.414435004853726	0.0481866637210007
SEC61A1	0.379137711665018	0.0232287095088901
HSP90B1	0.351924402739359	0.00886257554647448
USP31	0.349641961180969	0.0366932180402097
CALU	0.346337065498387	0.0437293919371684
TMSB10	0.344122233824892	0.0170777130178846
GAPDH	0.339690112195492	0.0191561641539018
LRP1	0.311748483086388	0.0232287095088901
EIF4G1	0.306637344924741	0.0447789888804968
ZFP36L1	0.265236634469688	0.0445112212460329
PPM1A	-0.333576466538726	0.0170777130178846
VPS13A	-0.349861726502702	0.0131873007548104
MGAT4A	-0.378932155119549	0.0233486320051801
TACC1	-0.386626379541474	0.047980932585511
ZRANB1	-0.4057913635913	0.0149462889482691
ESD	-0.428102244768157	0.0357557074858978
ACAA2	-0.457008895680877	0.016431500268858
PKHD1	-0.4624383398169	0.0289352307892795
KLF9	-0.464785307763336	0.00578162094102943
SMC5	-0.477449503056833	0.00754892103447511
FRMD3	-0.480218405123029	0.0322561483139933
ZNF189	-0.488229350702926	0.046731190643606
PTPRB	-0.535713179680496	0.0214807284742099
STXBP1	-0.558801587939771	0.0437293919371684
ESM1	-0.559964049127743	0.0425296515766892
KCNN3	-0.582558251822673	0.0201829878295162
PPP1R3E	-0.59549270702222	0.0491198828252648
SLC17A4	-0.597791570603741	0.0232287095088901
BTNL9	-0.605299364257515	0.0425681024635985
CHKA	-0.621277856707745	0.0203567870968191
LOC124906262	-0.621284137398769	0.00810318985993578
RNASE1	-0.634109864600786	0.0366932180402097
LDB2	-0.664362386060694	0.0124311392254767

LIN7A	-0.682132006493387	0.0166465340970221
EMCN	-0.725040816892281	0.0307382967609549
EDNRB	-0.725776697281214	0.00116949773452006
FANCC	-0.854085759526617	0.00116949773452006
ZNF471	-0.946197820031346	0.0252034728788225
LOC124909486	-1.01416198744606	0.030812801802097
ALDOB	-1.03986263133379	0.0497759758855165
KANK3	-1.12537846048365	0.033564165728732
TMEM233	-1.42476168080635	0.0209903083898389

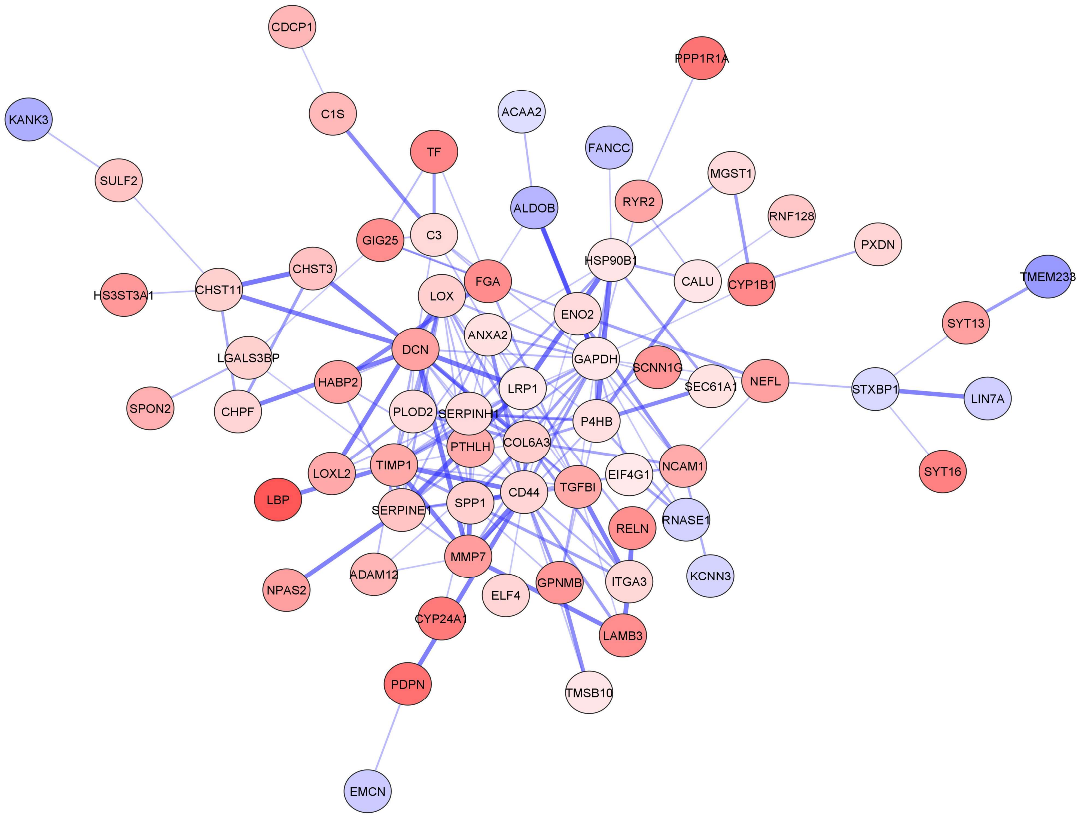

Supplementary Figure S1. PPI network diagram (STRING). Red indicates upregulated genes, and blue indicates downregulated genes. Node color saturation indicates a log fold-change. A thicker edge line corresponds to a higher confidence score.

Supplementary Table S2. Critical subnetworks extracted from the PPI network (MCODE).

Cluster	Score (Density*#Nodes)	Nodes	Edges	Genes
1	7.143	8	25	*MMP7*, *TGFBI*, *LOX*, *CD44*, *SPP1*, *SERPINE1*, *GADPH*, *DCN*
2	3.333	7	10	*ENO2*, *C3*, *FGA*, *GIG2*, *TF*, *NEFL*, *NCAM1*
3	3.200	6	8	*LRP1*, *HSP90B1*, *TIMP1*, *ANXA2*, *P4HB*, *COL6A3*
4	3.000	3	3	*CHST3*, *CHST11*, *CHPF*
5	3.000	3	3	*PLOD2*, *LOXL2*, *SERPINH1*

Supplementary Table S3. The top 20 hub genes per algorithm (Cytoscape's CytoHubba).

MCC	MNC	Degree	Closeness	Radiality	Stress	EPC
SPP1	*SPP1*	*SPP1*	*SPP1*	*SPP1*	*SPP1*	*SPP1*
LOX	*LOX*	*LOX*	*LOX*	*LOX*	*LOX*	*LOX*
P4HB	*P4HB*	*P4HB*	*P4HB*	*P4HB*	*P4HB*	*P4HB*
ITGA3	*ITGA3*	*ITGA3*	*ITGA3*	*HSP90B1*	*ITGA3*	*ITGA3*
HSP90B1	*HSP90B1*	*HSP90B1*	*HSP90B1*	*SERPINE1*	*HSP90B1*	*HSP90B1*
SERPINE1	*SERPINE1*	*SERPINE1*	*SERPINE1*	*ANXA2*	*SERPINE1*	*SERPINE1*
PLOD2	*PLOD2*	*ANXA2*	*ANXA2*	*ENO2*	*PLOD2*	*PLOD2*
ANXA2	*ANXA2*	*ENO2*	*ENO2*	*SEC61A1*	*ANXA2*	*ANXA2*
ENO2	*ENO2*	*GAPDH*	*GAPDH*	*GAPDH*	*ENO2*	*ENO2*
GAPDH	*GAPDH*	*COL6A3*	*COL6A3*	*COL6A3*	*GAPDH*	*GAPDH*
COL6A3	*COL6A3*	*LRP1*	*LRP1*	*LRP1*	*COL6A3*	*COL6A3*
LRP1	*LRP1*	*NEFL*	*NEFL*	*NEFL*	*LRP1*	*LRP1*
DCN	*DCN*	*DCN*	*DCN*	*DCN*	*DCN*	*DCN*
MMP7	*MMP7*	*MMP7*	*MMP7*	*MMP7*	*MMP7*	*MMP7*
TIMP1	*TIMP1*	*TIMP1*	*TIMP1*	*TIMP1*	*TIMP1*	*TIMP1*
NCAM1	*NCAM1*	*NCAM1*	*NCAM1*	*NCAM1*	*NCAM1*	*NCAM1*
CD44	*LAMB3*	*C3*	*C3*	*C3*	*GPNMB*	*GPNMB*
LOXL2	*CD44*	*CD44*	*CD44*	*CD44*	*CD44*	*CD44*
SERPINH1	*SERPINH1*	*SERPINH1*	*SERPINH1*	*SERPINH1*	*SERPINH1*	*SERPINH1*
TGFBI	*TGFBI*	*TGFBI*	*TGFBI*	*TGFBI*	*TGFBI*	*TGFBI*

Supplementary Table S4. Key transcription factors (TFs) of hub genes.

TF symbol	Description	P-value	Regulated genes
HIF1A	hypoxia inducible factor 1, alpha subunit (basic helix-loop-helix transcription factor)	5.34e−05	*GAPDH*, *LOX*, *SERPINE1*
CTNNB1	catenin (cadherin-associated protein), beta 1, 88kDa	0.000175	*CD44*, *MMP7*
TWIST1	twist basic helix-loop-helix transcription factor 1	0.000447	*CD44*, *TIMP1*
RUNX1	runt-related transcription factor 1	0.000584	*NCAM1*, *TIMP1*
SP1	Sp1 transcription factor	0.00071	*CD44*, *SERPINE1*, *SPP1*, *TIMP1*
CEBPA	CCAAT/enhancer binding protein (C/EBP), alpha	0.000949	*GAPDH*, *SPP1*
HDAC1	histone deacetylase 1	0.00183	*CD44*, *SPP1*
RELA	v-rel reticuloendotheliosis viral oncogene homolog A (avian)	0.00231	*NCAM1*, *SERPINE1*, *TIMP1*
NFKB1	nuclear factor of kappa light polypeptide gene enhancer in B-cells 1	0.00236	*NCAM1*, *SERPINE1*, *TIMP1*
STAT3	signal transducer and activator of transcription 3 (acute-phase response factor)	0.0071	*MMP7*, *TIMP1*
JUN	jun proto-oncogene	0.00779	*DCN*, *MMP7*

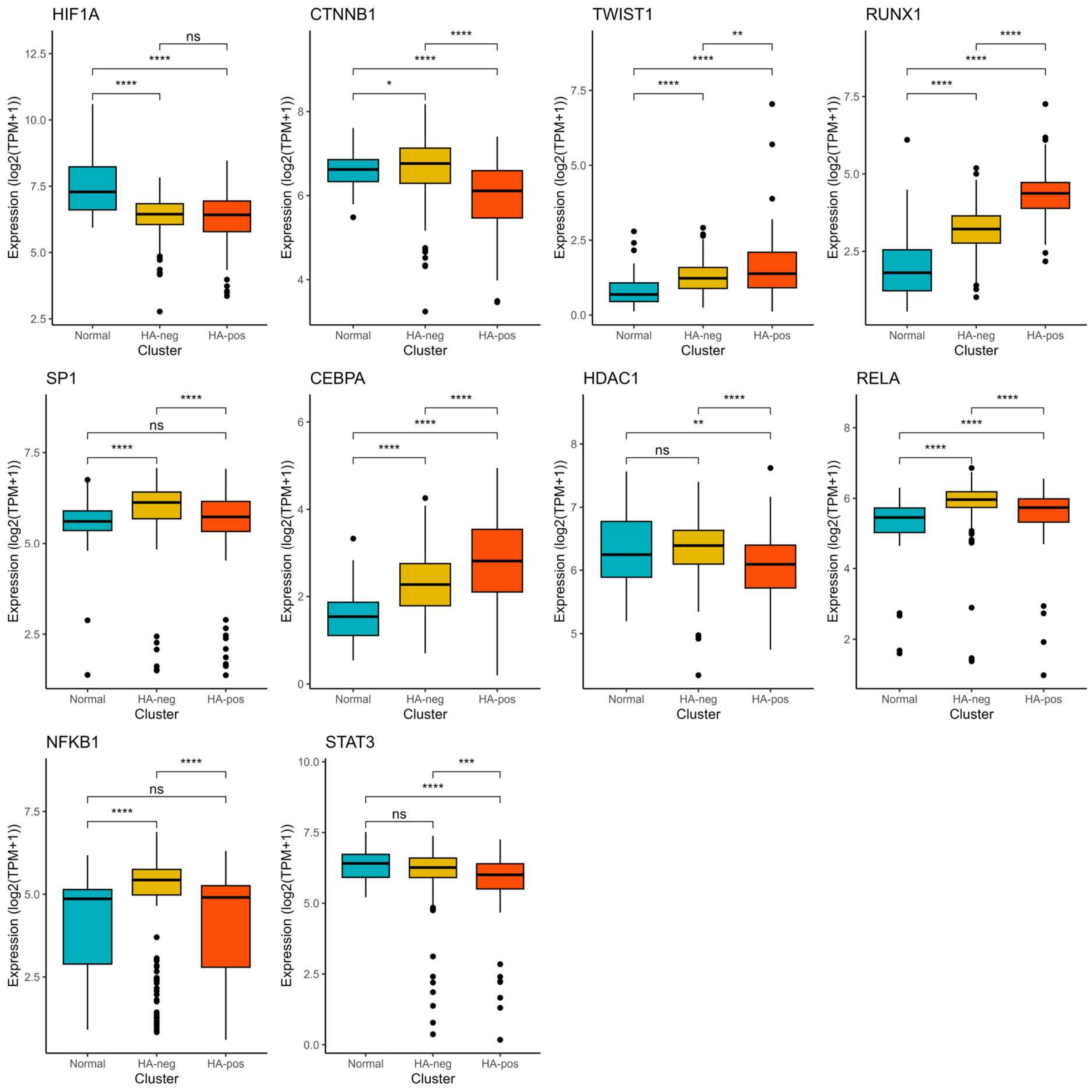

Supplementary Figure S2. Expression levels of transcription factors (TFs). The blue box denotes normal kidney tissue, the orange the HA-negative phenotype, and the red the HA-positive phenotype. The comparison between clusters was performed using the Mann–Whitney U-test. ****: $p \leq 0.0001$, ***: $p \leq 0.001$, **: $p \leq 0.01$, * $p \leq 0.05$, ns: $p > 0.05$.

Supplementary Table S5. Comparison of the Clinical characteristics between the discovery data set (HARCC) and TCGA datasets.

	HARCC				TCGA			
	**Hyaluronan-positive phenotype**		**Hyaluronan-negative phenotype**		**Hyaluronan-positive expression pattern**		**Hyaluronan-negative expression pattern**	
	**n (%)**		**n (%)**		**n (%)**		**n (%)**	
Samples	48		48		151		221	
Sex								
Male	31	(64.6)	24	(50)	118	(78.1)	123	(55.7)
Female	17	(35.4)	24	(50)	33	(21.9)	98	(44.3)
Age (Mean, range)	61.8	(41–82)	67.0	(36–86)	61.6	(26-90)	61.1	(34-90)
WHO/ISUP grade								
NA	0		0		1	(0.6)	2	(0.9)
1	2	(4.2)	8	(16.7)	0	(0)	10	(4.5)
2	22	(45.8)	22	(45.8)	34	(22.5)	118	(53.3)
3	10	(20.8)	9	(18.8)	66	(43.7)	82	(37.1)
4	14	(29.2)	9	(18.8)	50	(33.1)	9	(4.1)
Sarcomatoid change								
No	41	(85.4)	43	(89.6)	NA		NA	
Yes	7	(14.6)	5	(10.4)	NA		NA	
Stage*								
NA	0		0		2	(1.3)	0	
I	16	(33.3)	24	(50.0)	40	(26.5)	133	(60.2)
II	9	(18.8)	8	(16.7)	16	(10.6)	22	(10.0)
III	12	(25.0)	7	(14.6)	53	(35.1)	44	(19.9)
IV	11	(22.9)	9	(18.8)	40	(26.5)	22	(10.0)
Metastasis at diagnosis								
NA	0		0		3	(2.0)	14	(6.3)
M0	37	(77.1)	39	(81.3)	109	(72.2)	186	(84.2)
M1	11	(22.9)	9	(18.7)	39	(25.8)	21	(9.5)
Disease-related death								
NA	0		0					
No	28	(58.6)	35	(72.9)	81	(53.6)	168	(76.0)
Yes	20	(41.2)	13	(27.1)	70	(46.4)	53	(24.0)

NA: Not available

*HARCC stage is defined using cTNM and TCGA stage is based on pTNM

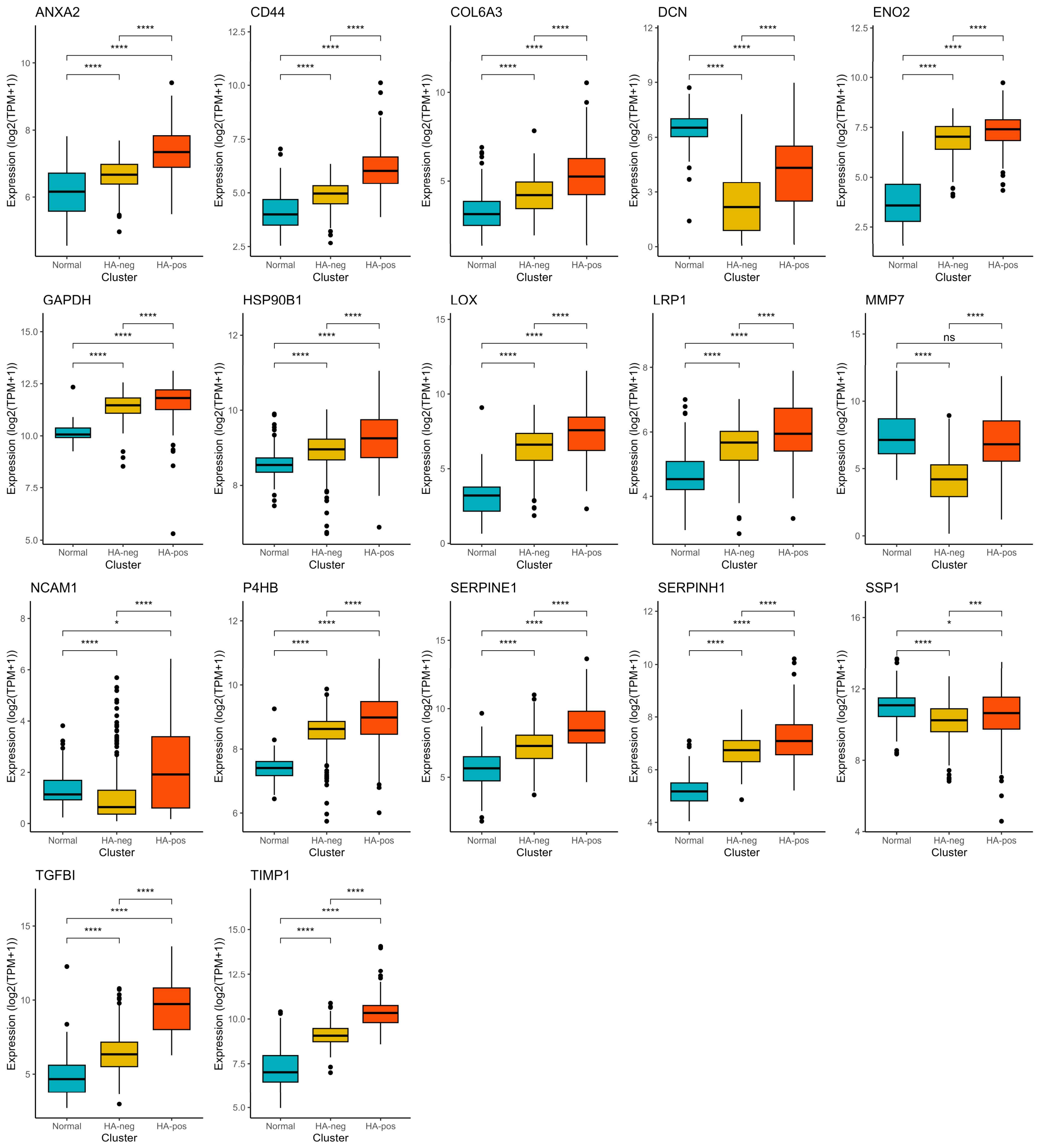

Supplementary Figure S3. Expression levels of hub genes in TCGA-KIRC material. The blue box denotes normal kidney tissue, the orange the HA-negative phenotype, and the red the HA-positive phenotype. The comparison between clusters was performed using the Mann–Whitney U-test. ****: $p \leq 0.0001$, ***: $p \leq 0.001$, **: $p \leq 0.01$, * $p \leq 0.05$, ns: $p > 0.05$.

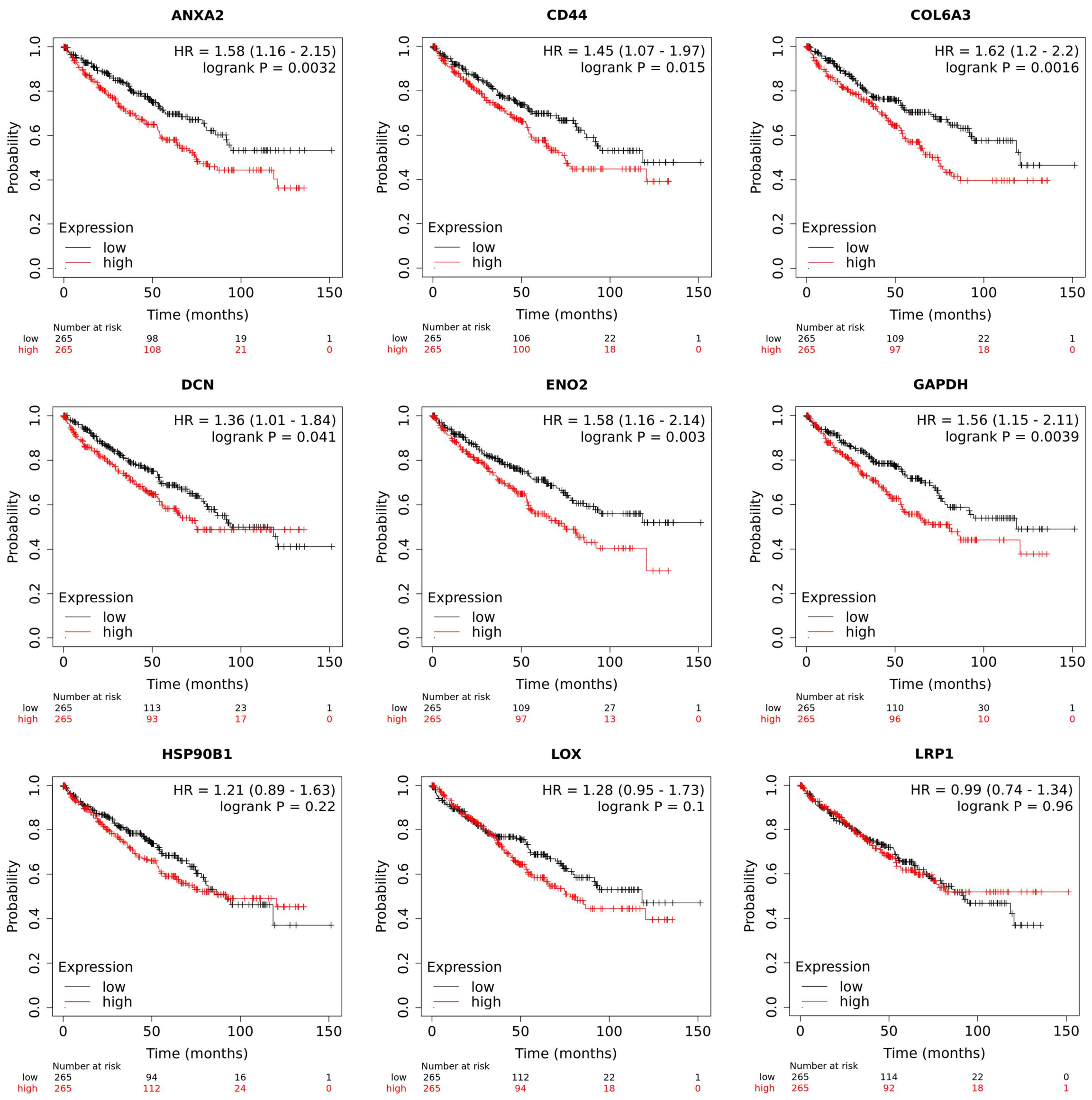

Supplementary Figure S4. Survival plots (OS) of hub genes, part 1.

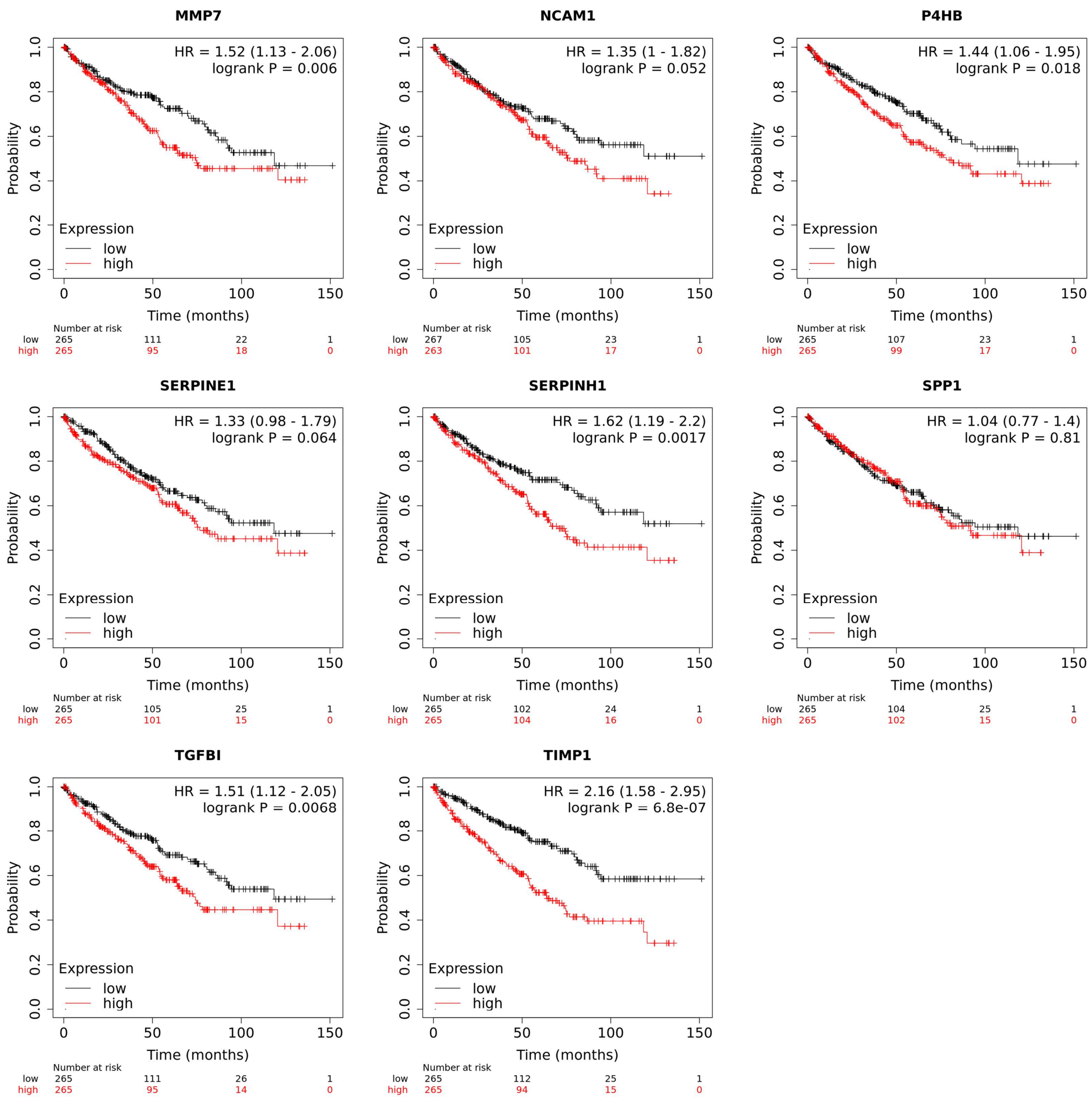

Supplementary Figure S5. Survival plots (OS) of hub genes, part 2.

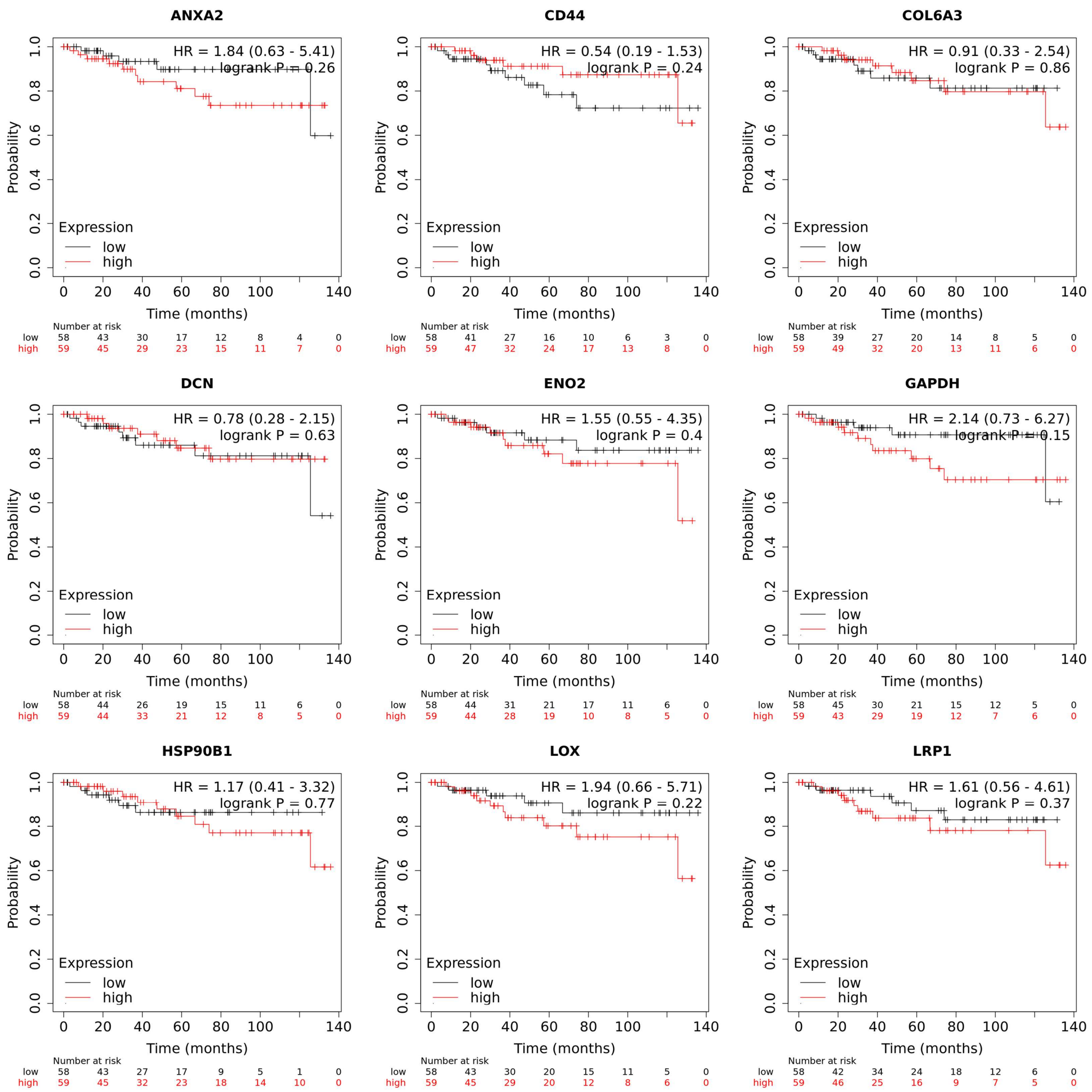

Supplementary Figure S6. Survival plots (DFS) of hub genes, part 1.

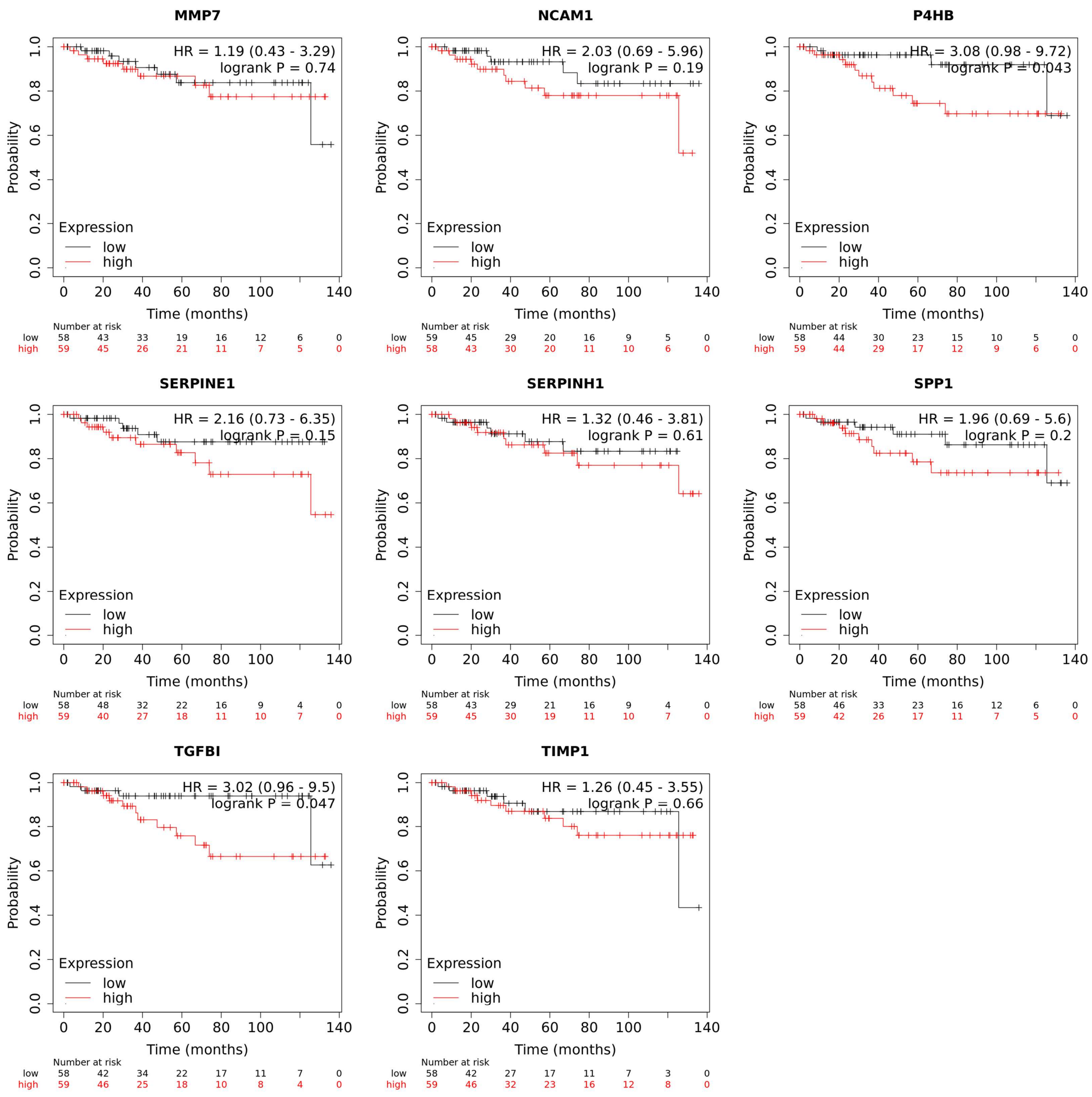

Supplementary Figure S7. Survival plots (DFS) of hub genes, part 2.
